# Feasibility of a Condensed EEG Curriculum Using a Procedural Based Learning Model

**DOI:** 10.1177/15500594261430384

**Published:** 2026-03-05

**Authors:** Alexandria Valdrighi, Susannah Cornes, Vanja Douglas, Audrey Foster-Barber, Ernesto Gonzalez-Giraldo

**Affiliations:** 1 Department of Neurology, 8785 University of California San Francisco, San Francisco, CA, USA

**Keywords:** EEG, medical education, epilepsy, Fitts-Posner model, resident learning

## Abstract

**Background:**

Neurology residency time constraints limit requisite EEG training, contributing to discomfort with this core skill. EEG interpretation is a procedural skill, and educational models utilizing cognitive and procedural elements may increase education efficacy and efficiency. We applied the Fitts-Posner procedural model to create a condensed EEG curriculum and assessed feasibility and efficacy.

**Methods:**

We identified EEG education barriers then designed a curriculum including asynchronous didactics, EEG reading/staffing, and report writing. The curriculum was incorporated 2023-2024 into a 2-week (adult neurology) or 4-week (child neurology) epilepsy rotation. We evaluated EEG interpretation knowledge and confidence before, after and following a 3-month delay and solicited feedback.

**Results:**

Primary barriers to EEG education were insufficient practice and clinical responsibility. Ninety percent (19/21) of residents completed the curriculum. Residents identified reading/staffing EEGs and writing reports as instrumental curriculum components. Compared with pre-rotation, confidence and knowledge increased immediately post-rotation and following a 3-month delay but were lower at 3-months than immediately post-rotation. Child neurology residents had higher confidence than adult residents on post and 3-month surveys despite no knowledge differences.

**Conclusions:**

A condensed EEG curriculum using a procedural learning model was associated with improved confidence and knowledge after a 3-month delay. Confidence and knowledge were lower at 3-months than immediately post-rotation, indicating the need for reinforcing activities. Confidence was higher for child neurology residents with two extra rotation weeks suggesting additional training may also enhance confidence gains. Asynchronous didactics maximized efficiency, but residents valued reading/staffing EEGs and writing reports, highlighting the importance of sociocultural approaches.

## Introduction

Neurologists frequently interpret electroencephalograms (EEGs) but often lack appropriate training.^1,2^ A survey of graduating neurology residents found that only 37% of adult and 67% of child neurology residents felt confident autonomously interpreting EEGs.^3–5^ Common reasons for inadequate EEG training are insufficient experience and didactics.^5–8^

Eight weeks of EEG instruction is proposed as the minimum time for gaining proficiency.^8,9^ However, this is often not feasible for programs juggling competing educational competencies. Optimized instruction methods need to be defined to maximize efficiency. EEG education literature suggests that condensed virtual modules and reading practice are effective.^8–18^ However, there are few studies utilizing a combination of these approaches and those that do are implemented over an extended timeframe.^16,17^ Additionally, there is limited assessment of condensed EEG curriculum's long-term efficacy.

Time-based evaluation metrics for EEG education likely contribute to nonoptimized training; instead, tailoring curriculum to defined competency levels may be more beneficial.^4,5,19^ Surgical specialties have made similar paradigm shifts providing a framework for this adaptation.^19–22^ The Fitts-Posner model is one procedural approach which outlines sequential advancement through stages of skill acquisition. Learners progress from understanding procedural steps to performing the task with minimal cognitive load.^19^ Procedural-based learning models have not been applied to EEG instruction and may allow for enhanced skill advancement within shorter rotations.

We assessed the feasibility and short-and-long-term efficacy of a condensed two-or-four-week EEG education program designed using the Fitts-Posner model.

## Methods

Prior to curriculum implementation, 67% (12/17) of PGY4-5 adult and child neurology residents at the University of California, San Francisco (UCSF) completed a needs assessment evaluating EEG education barriers and preferred instruction methods (Appendix A).^7^ Needs assessment participants had completed epilepsy rotations and did not participate in our curriculum. Prior to our intervention, EEG instruction for adult residents included a pediatric epilepsy rotation without EEG reading requirements and for child neurology residents an EEG reading rotation; neither included formal didactics. We incorporated our curriculum into the 2023-2024 2-week pediatric epilepsy rotation for PGY-3 adult residents and the 4-week EEG rotation for PGY-4 child neurology residents.

**Figure 1.**
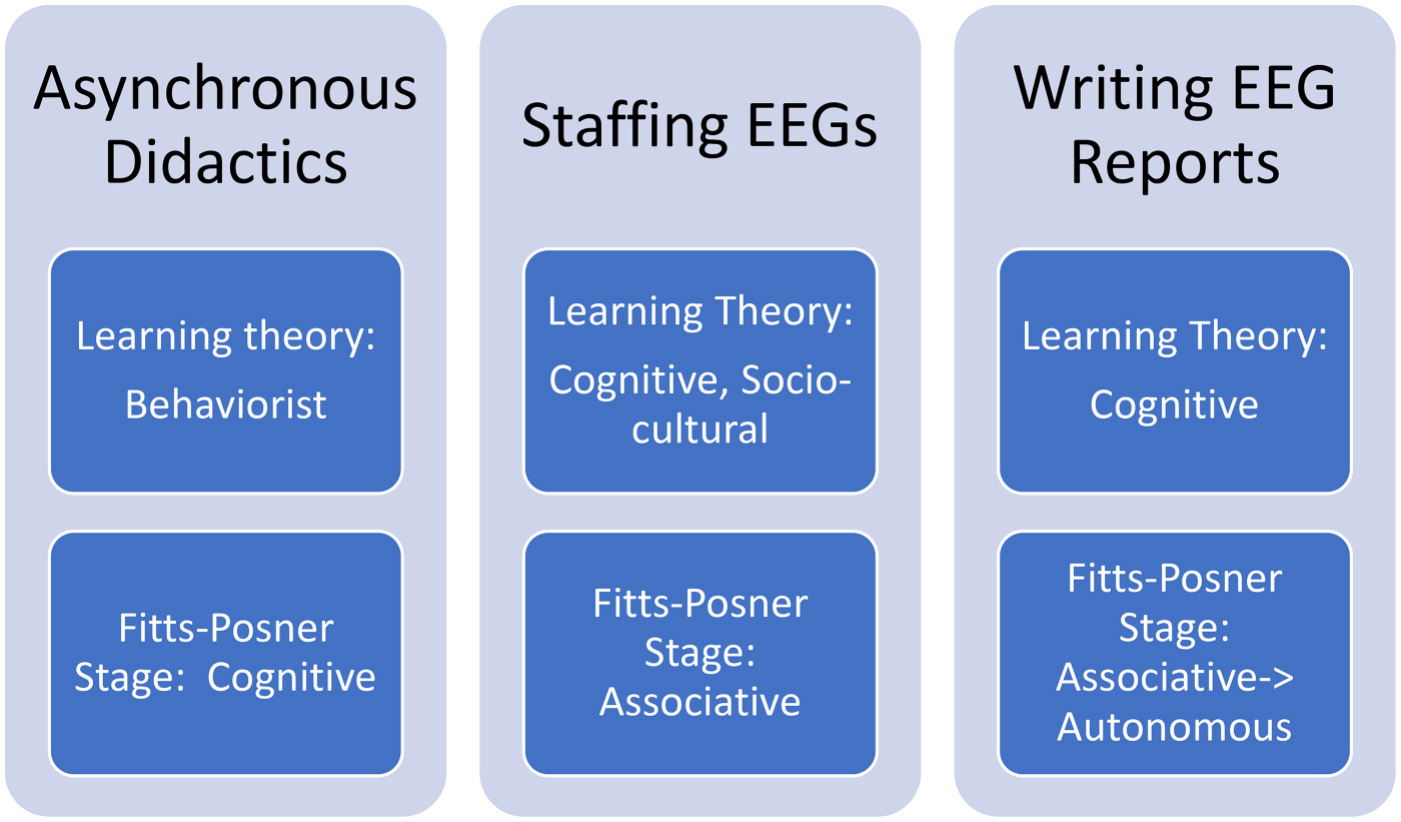
Alignment of curriculum components to Fitts-Posner stages and related learning theories. Asynchronous didactics included three 30–60 slide PowerPoint presentations that targeted the Fitts-Posner cognitive stage and behaviorist learning theory by teaching a stepwise framework with which to approach EEG interpretation. Staffing EEGs targeted the associative stage of the Fitts-Posner model and cognitive learning theory by allowing application of the previously learned approach to EEG interpretation. It also incorporated sociocultural learning theory through interaction with epilepsy attendings. Writing EEG reports incorporated cognitive learning theory and served as a bridge to the autonomous Fitts-Posner stage by requiring independent synthesis of information.

We applied Kern's six steps for curriculum development and targeted instructional techniques to stages of the Fitts-Posner model incorporating behaviorist, sociocultural and cognitive elements (Figure 1).^19,22,23^ The Fitts-Posner model describes a process of skill acquisition that moves learners from beginner to advanced stages. The model includes cognitive, associative and autonomous phases. In the cognitive stage, learners are introduced to the basic concepts and steps involved in performing a task. In the associative stage, learners practice performing the task which requires significant conscious effort. Learners then eventually progress to the autonomous stage where completing the task becomes natural and can be done with minimal cognitive load.^19^ Our curriculum advanced participants through cognitive and associative phases and allowed them to reach the autonomous stage with ongoing practice. Although EEG reading lacks the physical application of many procedural tasks, it is a process of skill acquisition that can benefit from similar learning approaches. The use of the Fitts-Posner model structures the curriculum to direct each didactic component to a specific stage of competency which allows more targeted focus of educational tools.

Curriculum objectives were based on ACGME EEG reading milestones and competencies [Table 1].^24,25^ We created three asynchronous 30–60 slide PowerPoint presentations entitled *The Normal EEG, The Abnormal EEG and Using EEG Software and Report Creation* to cover these objectives. The lectures covered both pediatric and adult EEG findings and the abnormal EEG lecture incorporated critical care ICU EEG topics. Neonatal EEG was not covered in the didactics. Residents read EEGs and wrote clinical reports for a minimum of 2 routine EEGs per day. EEGs were staffed daily during a dedicated staffing session with the epileptologist covering the inpatient service. Epileptologists rotated on a weekly basis. The *Using EEG Software and Report Creation* didactic provided an outline for structuring EEG reports. Using this framework and provided templates, EEG reports were drafted by residents prior to staffing the EEGs. The reports were then reviewed with the on-service epileptologist for feedback and corrections during the staffing session.

**Table 1.** Asynchronous Didactic Learning Objectives.

Identify cases where EEG is an appropriate diagnostic tool.
Recognize normal EEG background features in children and adults including common background variants.
Recognize normal sleep architecture on EEG.
Recognize common EEG artifacts.
Describe patterns of status epilepticus in children and adults.
Recognize common epileptiform and non-epileptiform EEG abnormalities in children and adults.
Feel comfortable using EEG reading software and generating an EEG report.
Recognize when to seek help from an epileptologist.

We utilized surveys adapted from the literature to assess residents’ EEG reading confidence and knowledge before and after the curriculum and three months after rotation completion (Appendix A).^7,10,11^ An average Likert confidence score was calculated for each participant based on their reported ability to identify defined EEG patterns. A knowledge score was calculated as percent of correctly answered EEG reading questions. Residents received answers after the 3-month survey. We solicited curriculum feedback.

We compared knowledge and confidence scores at each time point using the Wilcoxon matched pair signed rank test and number of EEGs read, knowledge and confidence between adult and child neurology residents using the Wilcoxon test. Alpha was set at .05 and statistical tests performed in Stata.

Our procedures were reviewed by the UCSF Institutional Review Board (November 1, 2022; IRB number 22-37362) and determined to be exempt. Need for ethics approval and participant consent for the collection, analysis and publication of anonymized data were waived. Resident participation was optional, and consent implied by survey completion. Survey data was anonymized prior to review.

## Results

Nineteen of twenty-one residents completed the curriculum and assessment surveys (90%). Two residents were excluded for not completing the surveys.

**Figure 2.**
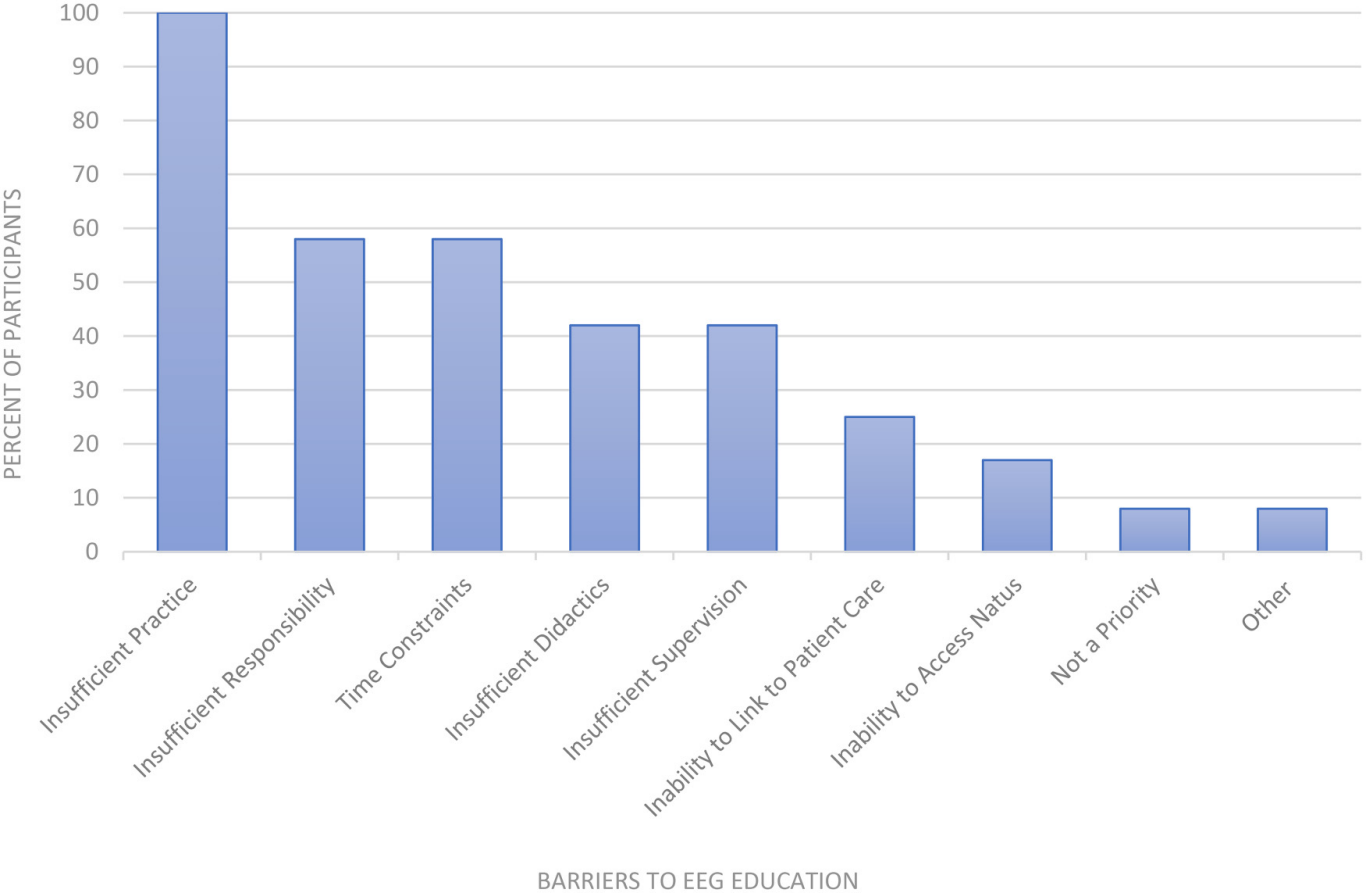
Barriers to EEG education identified on needs assessment survey. Percent of needs assessment participants that selected each option as a barrier to EEG education prior to our intervention. Participants were allowed to select from a list of the above EEG education barriers and could choose multiple barriers.

Residents identified limited EEG reading practice and insufficient clinical responsibility as top education barriers on the needs assessment prior to curriculum implementation (Figure 2). One hundred percent of residents on the needs assessment reported a combination of didactics and EEG review as their ideal method of EEG instruction.

**Figure 3.**
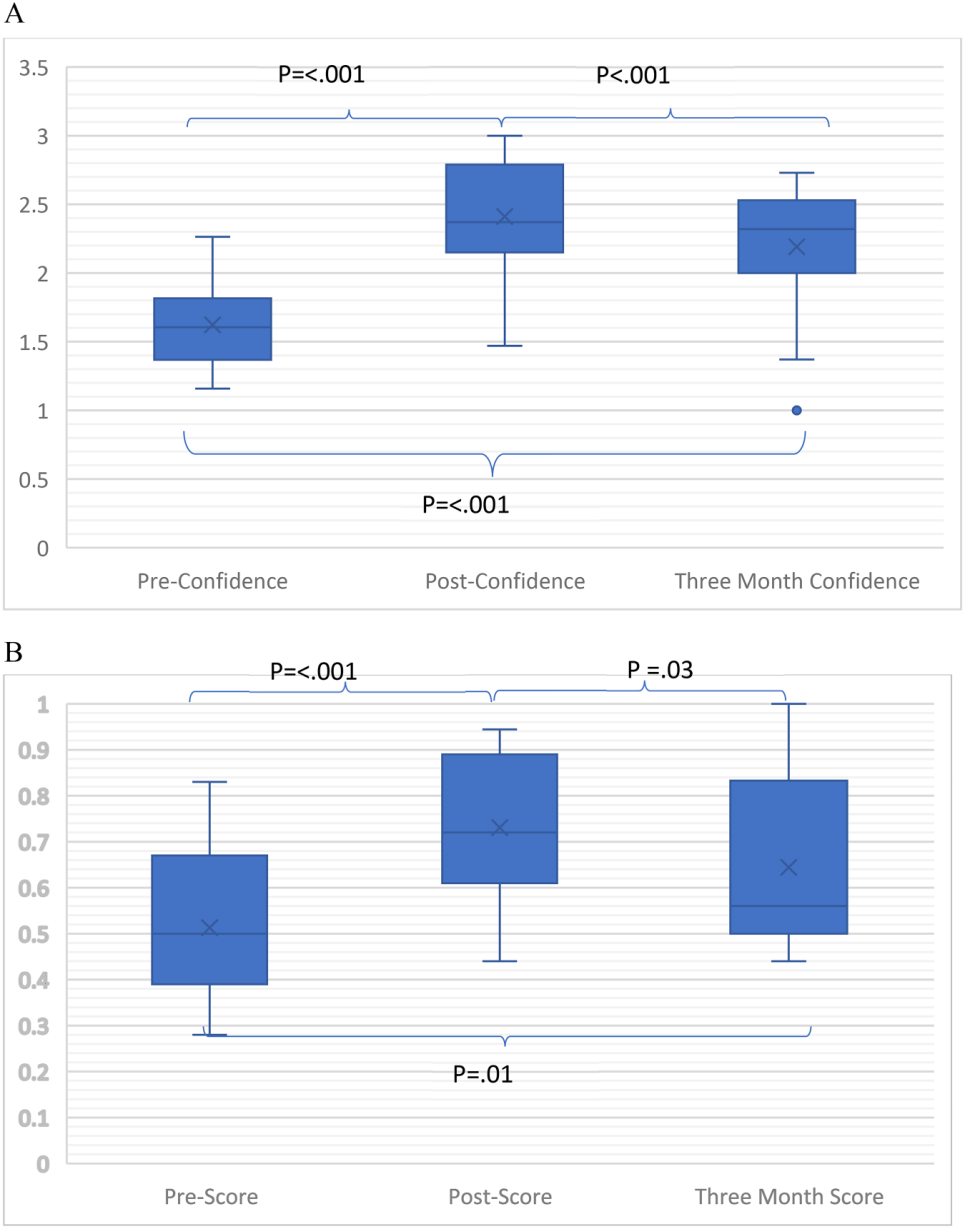
(A) Comparison of median pre-curriculum, post-curriculum and three-month post curriculum confidence scores. Confidence score was calculated as an average Likert score (1-I cannot recognize these features to 4-I always recognize these features independently) of responses to questions on identifying EEG features. Median[IQR] post [2.37(2.15–2.79)] and three month [2.32(2–2.53)] confidence scores were higher than pre-curriculum scores [1.61(1.37–1.81). Median three-month confidence scores were lower than post curriculum. (B) Comparison of median pre-curriculum, post-curriculum and three-month post curriculum knowledge scores. Knowledge score was calculated as percent of EEG reading questions answered correctly. Median[IQR] post [0.72(0.61–0.89) and three-month knowledge [0.56(0.50–0.83)] scores were higher than pre-curriculum [0.50(0.39–0.67)]. Median three-month knowledge scores were lower than post curriculum.

Residents read a median of 10 [IQR 4-20] EEGs and wrote 5 [4-10] reports during the rotation. Most participants had not read EEGs [0 (0-0)] or written [0 (0-0)] reports previously. 42% (8/19) of residents had read EEGs prior to the rotation and 11% (2/19) had written reports. Compared with pre-rotation, residents had higher EEG interpretation knowledge and confidence post-rotation (*p* < 0.001) and after a three-month delay (*p* < 0.001; Figure 3A, B). However, confidence (*p* < 0.001) and knowledge (*p* = 0.03) were both lower at three months compared with immediately post-curriculum (Figure 3A, B).

**Figure 4.**
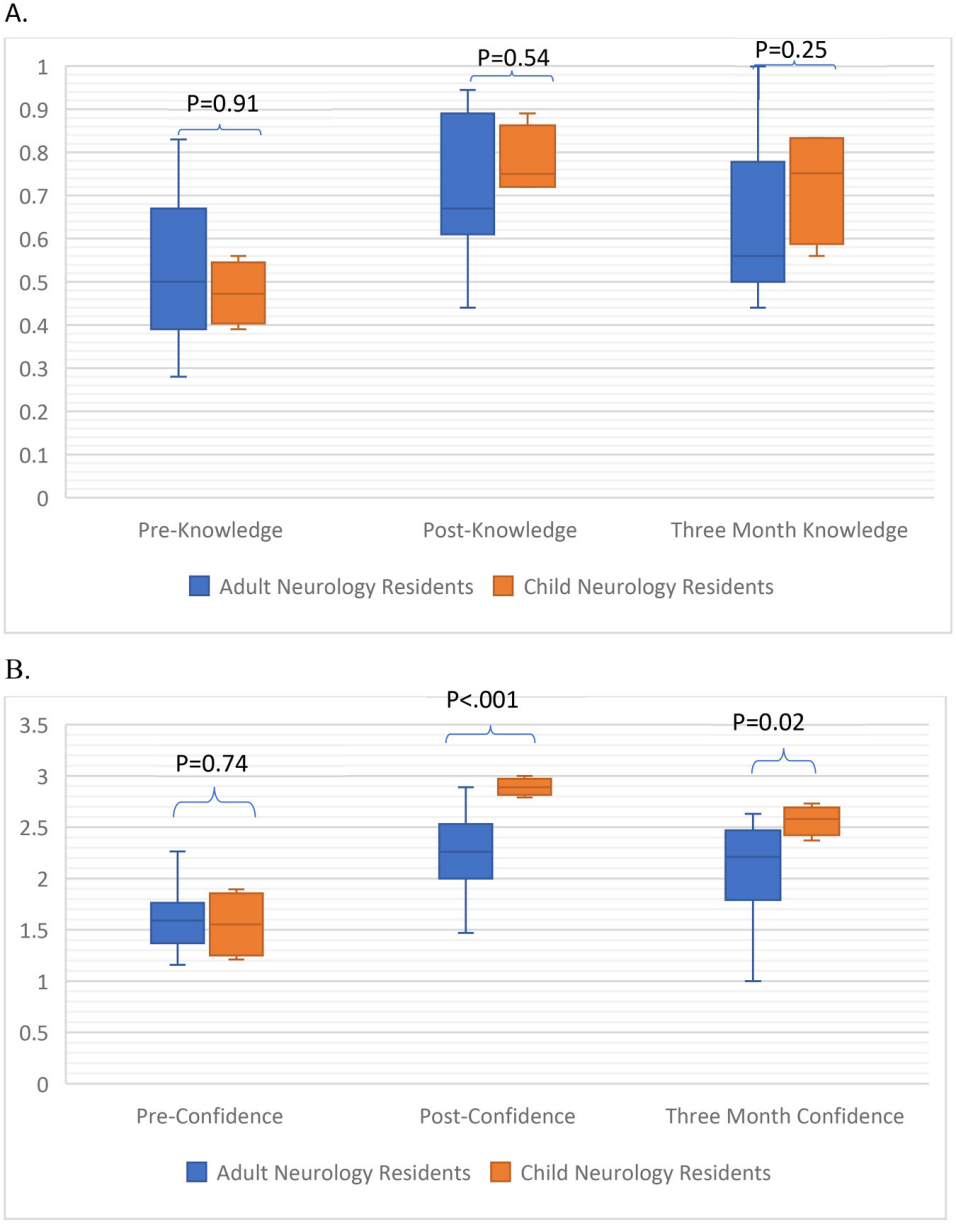
(A) Comparison of median pre-curriculum, post-curriculum and three-month post knowledge scores between child and adult neurology residents. There was no difference in median[IQR] knowledge scores between groups at any timepoint [child pre-0.47(0.42–0.53), post-0.75(0.72–0.83), 3-month post-0.67(0.61–0.89); adult pre-0.5(0.39–0.67), post-0.67(0.61–0.89), 3-month post-0.56(0.50–0.78)]. (B) Comparison of pre-curriculum, post-curriculum and three-month post median confidence scores between child and adult neurology residents. Child neurology residents had higher median confidence scores following the curriculum and after a three-month delay [child pre-1.55(1.29–1.82), post-2.89(2.84–2.95), 3-month post-2.58(2.48–2.66); adult pre-1.60(1.47–1.79) post-2.26(2–2.53), 3-month post-2.21(1.79–2.47).

Child neurology residents read (*p* = 0.01) and wrote (*p* < 0.001) reports for more EEGs [31 (23–46)] than adult residents [read 6 (4–15); reports 5 (4–6)]. There was no difference in knowledge between groups at any point (Figure 4A). Despite no difference in pre-curriculum confidence (*p* = 0.74), child neurology residents had higher confidence post-curriculum (*p* < 0.001) and after three-months (*p* = 0.02) (Figure 4B).

Residents reported reading/staffing EEGs and writing reports as most beneficial. On 3-month follow-up, 89% (17/19) revisited the asynchronous didactics and 47% (9/19) continued reviewing EEGs.

## Discussion

Residents participating in our curriculum had improved confidence and knowledge immediately following their rotation and after a 3-month delay, suggesting the feasibility and efficacy of condensed EEG education. Application of a procedural model to focus curriculum on specific competency levels likely contributed to the success and could be used as a framework for designing EEG education within residency time constraints. Similar findings are demonstrated in surgical learning with competency focused advancement.^19–21^ Additionally, most residents revisited educational materials and continued reviewing EEGs post-rotation allowing for ongoing progression.

Prior barriers to EEG education included limited practice and responsibility for reading EEGs which is consistent with the past literature.^1–7^ We utilized self-guided didactics to allow for increased EEG review during a short rotation block. Asynchronous didactics are effective and can be expanded in EEG training to maximize efficiency and flexibility, increase time for EEG review and facilitate standardized learning experiences.^9–12^^,15^-Additionally, residents can revisit materials for knowledge consolidation as many did in our study.^9–12^^,15^

Residents viewed EEG reading and report drafting as the most efficacious parts of our intervention. It has previously been demonstrated that didactics without clinical application have limited utility which corresponds with our results.^13^ Writing reports is not discussed in past studies and may promote substantial learning through independent synthesis of information that links to the autonomous Fitts-Posner stage.^8–18^ Residents also highly valued staffing EEGs illustrating the importance of applying a sociocultural lens to EEG education.

Confidence and knowledge decreased three months after the rotation indicating further decrement may have continued with time. Longitudinal reinforcing activities are needed in combination with condensed dedicated training to prevent loss of learning gains. Additionally, child neurology residents who read more EEGs than their adult counterparts had higher post and three-month confidence scores despite no difference in knowledge. Additional EEG reading practice may also be needed for residents to achieve confidence in their interpretations. Future directions include incorporating of longitudinal activities and assessing for sustained gains.

Limitations to our study include a small sample size from a single site which limits finding generalizability. Additionally, we assessed knowledge utilizing a short survey format and EEG screenshots rather than review of EEG files. This may have limited our ability to fully capture the extent of participant knowledge. It may also have contributed to the lack of higher knowledge scores demonstrated by child neurology residents compared to their adult counterparts.

## Conclusions

We demonstrate the feasibility and efficacy of a condensed EEG curriculum applying a procedural based learning model. However, longitudinal reinforcing activities are still needed to prevent skill decrement and may facilitate increased confidence gains. Asynchronous didactic use can facilitate time for EEG reading practice and report writing which are most valued by residents.

## Supplemental Material

sj-docx-1-eeg-10.1177_15500594261430384 - Supplemental material for Feasibility of a Condensed EEG Curriculum Using a Procedural Based Learning Model Supplemental material, sj-docx-1-eeg-10.1177_15500594261430384 for Feasibility of a Condensed EEG Curriculum Using a Procedural Based Learning Model by Alexandria Valdrighi, Susannah Cornes, Vanja Douglas, Audrey Foster-Barber and Ernesto Gonzalez-Giraldo in Clinical EEG and Neuroscience
