# Supplementary material for: Feasibility of a Condensed EEG Curriculum Using a Procedural Based Learning Model
Source: Clin EEG Neurosci. 2026 Mar 5;57(4):351–7. doi: 10.1177/15500594261430384 (PMC13242535; doi:10.1177/15500594261430384)
Supplement: sj-docx-1-eeg-10.1177_15500594261430384 - Supplemental material for Feasibility of a Condensed EEG Curriculum Using a Procedural Based Learning Model [file sj-docx-1-eeg-10.1177_15500594261430384.docx]

Title: Appendix A.

**Description: Needs assessment, pre, post and 3-month post surveys utilized.**

NEEDS ASSESSMENT SURVEY

Start of Block: Default Question Block

Q1  Please enter your full name. This information will be used only to compare pre- and post-survey results and will be re-coded for anonymity prior to analysis.

________________________________________________________________

End of Block: Default Question Block

Start of Block: The next questions will assess your interest in EEG reading

Q43 The next questions will assess your interest in EEG reading.

Q2 Are you planning to pursue additional fellowship training in epilepsy and/or neurophysiology?

- Yes (1)
- No (2)
- Unsure (3)

Q3 How important is it to learn EEG reading during neurology residency?

- Not at all important (1)
- Slightly important (2)
- Moderately important (3)
- Very important (4)
- Extremely important (5)

Q4 Describe your level of agreement with the following statement: "Learning to read EEGs during neurology residency is only important if you are pursuing a fellowship in epilepsy and/or neurophysiology. "

- Strongly agree (1)
- Agree (2)
- Neutral (3)
- Disagree (4)
- Strongly disagree (5)

End of Block: The next questions will assess your interest in EEG reading

Start of Block: The next questions will assess your current comfort with EEG reading

Q44 The next questions will assess your experience with EEG resident education.

Q5 Which rotations have you participated in that included EEG education and/or EEG reading practice? Please check all that apply.

- Adult Epilepsy Monitoring Unit (1)
- Pediatric Epilepsy Monitor Unit (2)
- VA Epilepsy Rotation (3)
- Pediatric EEG reading rotation (4)
- Others (please specify) (5) __________________________________________________

Q6 Which additional EEG focused rotations do you plan to participate in before graduating that you have not yet completed? Please check all that apply.

- Adult Epilepsy Monitoring Unit (1)
- Pediatric Epilepsy Monitor Unit (2)
- VA epilepsy rotation (3)
- Pediatric EEG reading rotation (4)
- Others (please specify) (5) __________________________________________________

Q7 How many EEGs do you estimate you have read during residency? "Read" means reviewed an EEG in its entirety but does not need to include drafting an EEG report.

________________________________________________________________

Q8 How many EEG reports do you estimate you have written during residency?

________________________________________________________________

Q9 What are the forms of EEG didactics you have participated in? Check all that apply

- Formal didactics given during a specific epilepsy and/or EEG focused rotation (1)
- Formal didactics given during a non-epilepsy and/or EEG focused rotation (2)
- Formal EEG didactics during physicianship (3)
- Formal EEG didactics during adult Thursday outpatient education (4)
- Formal EEG didactics during pediatric neurology Thursday afternoon conference (5)
- Formal EEG didactics during Wednesday afternoon conference (6)
- Informal teaching during a specific epilepsy and/or EEG focused rotation (7)
- Informal teaching given during a non-epilepsy and/or EEG focused rotation (8)
- No didactics (9)
- Other (please specify) (10) __________________________________________________

Q10  Of the EEG education you have completed so far, which have been the most helpful?

________________________________________________________________

Q11 What do you consider barriers to learning EEG? Check all that apply

- Insufficient exposure to EEG reading (1)
- Insufficient amount of EEG didactics and/or educational materials (2)
- Suboptimal education/supervision from attendings and/or fellows (3)
- Not a priority since I am not pursuing an EEG/epilepsy career (4)
- Inability to link EEG learning to direct patient care (ie reading EEGs and caring for epilepsy patients at different times) (5)
- Insufficient responsibility to read EEGs and formulate reports during EEG rotations (6)
- Time constraints due to high patient volume (7)
- Not knowing how to use EEG reading software (Natus) (8)
- Other (please specify) (9) __________________________________________________

Q12 Of the barriers selected above, which has been the most significant barrier?

________________________________________________________________

Q13 What solutions would you propose to the above barriers you have experienced?

________________________________________________________________

Q14 What would be your preferred method for learning EEGs?

- Self-guided lecture/module only (1)
- In-person lecture/module only (2)
- EEG reading practice only (3)
- Self-guided lecture/module + EEG reading practice (4)
- In-person lecture/module + EEG reading practice (5)
- Other (please specify) (6) __________________________________________________

Q15 Please provide us any additional suggestions or comments on how to improve the current EEG education.

________________________________________________________________

End of Block: The next questions will assess your experience with EEG resident education

Start of Block: Please tell us a little bit about yourself,

Q45 Please tell us a little bit about yourself.

Q16 What is your current gender identify? Please select all that apply.

- Male/Man (1)
- Female/Woman (2)
- Non-binary (3)
- Prefer not to say (4)
- Other (please specify) (5) __________________________________________________

Q17 What is your ethnic/racial identity? Please select all that apply.

- Black/African American (1)
- Latinx or Hispanic (2)
- Asian (3)
- Pacific Islander or Native Hawaiian (4)
- White/Caucasian (5)
- Indigenous/Native American (6)
- Middle Eastern/North African (7)
- Prefer not to say (8)
- Other (please specify) (9) __________________________________________________

Q18  Are you an adult neurology or child neurology resident?

- Adult neurology (1)
- Child neurology (2)
- Other (please specify) (3) __________________________________________________

Q19 What is your level of training?

- PGY-1 (1)
- PGY-2 (2)
- PGY-3 (3)
- PGY-4 (4)
- PGY-5 (5)
- Other (please specify) (6)

Q20 What area/subspecialty of neurology do you plan to practice in for your future career (i.e. general outpatient neurology, neurohospitalist, neuromuscular, epilepsy etc.). ?

________________________________________________________________

End of Block: Please tell us a little bit about yourself,

Pre-Rotation EEG Reading Survey

Start of Block: Default Question Block

Q1 Please enter your full name. This information will be used only to compare pre- and post-survey results and will be re-coded for anonymity prior to analysis.

________________________________________________________________

| Page Break |  |
| --- | --- |

Q52 The next questions will assess your current ability to read EEGs.

End of Block: Default Question Block

Start of Block: The next questions will assess your current ability to read EEGs.

Q2 A 33-year-old woman presents to the emergency room after having a seizure. Your workup includes a routine EEG prior to the patient leaving the emergency room. Which of the following statements is true?

- **The presence of focal interictal epileptiform discharges on EEG has a high positive predictive value for epilepsy (1)**
- The absence of focal interictal epileptiform discharges on EEG will rule out epilepsy (2)
- If the event concerning for seizure is not captured on EEG, the test will not help predict the patient's risk of epilepsy (3)
- The frequency of focal interictal epileptiform discharges on EEG does not have significant prognostic value (4)

Q3 When is the rhythm outlined in the box below best seen on EEG?


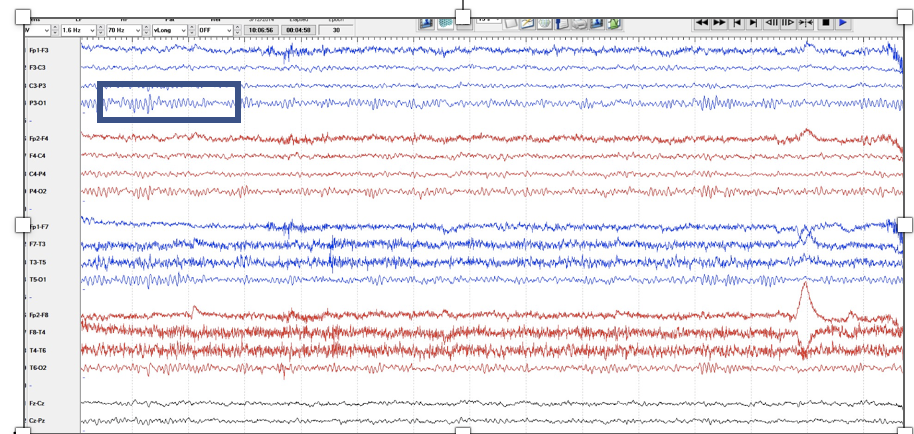


- During drowsiness (1)
- During thoughts of moving the contralateral arm (2)
- **During wakefulness with eyes closed** (3)
- During strenuous mental tasks (4)
- During sleep (5)

Q5 What is the patient most likely doing during the EEG segment below?


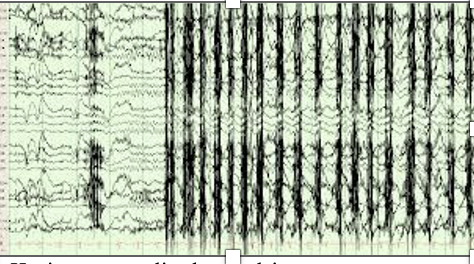


- Having a generalized convulsion (1)
- Walking around the room (2)
- **Eating a snack** (3)
- Shaking their head from side to side (4)

Q15 What is the likelihood that this EEG pattern is consistent with non-convulsive status epilepticus (NCSE) in a patient with no clinical correlate to this pattern?

  
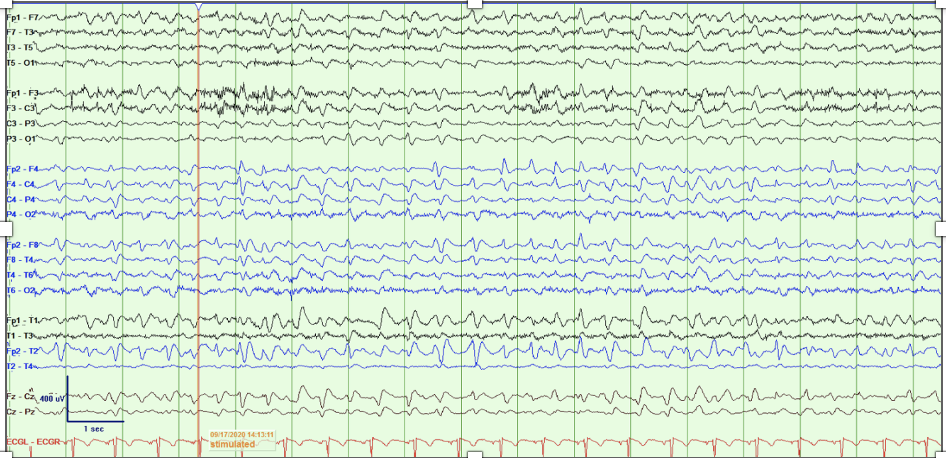


- **The EEG is probably not consistent with NCSE because the rhythmic discharges are slower than 2.5hz and do not evolve** (1)
- This EEG is probably not consistent with NCSE because the rhythmic discharges are triphasic appearing (2)
- This EEG is probably NCSE because there are rhythmic discharges greater than 2.5hz (3)
- This EEG is probably NCSE because this pattern emerged after stimulation of the patient (4)

Q8 In the displayed EEG, what is the box over the right temporal region highlighting?


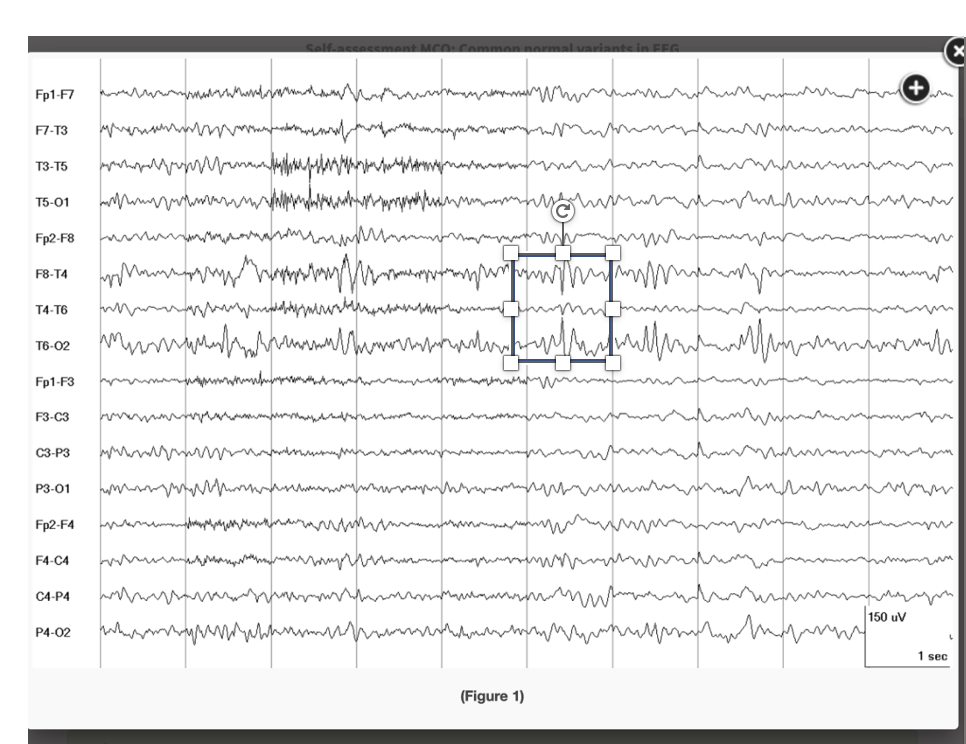


- Mu rhythm (1)
- **Wicket Spike** (2)
- Temporal Spike (3)
- POSTs (4)

Q9 How old is the patient whose normal awake EEG is pictured here?


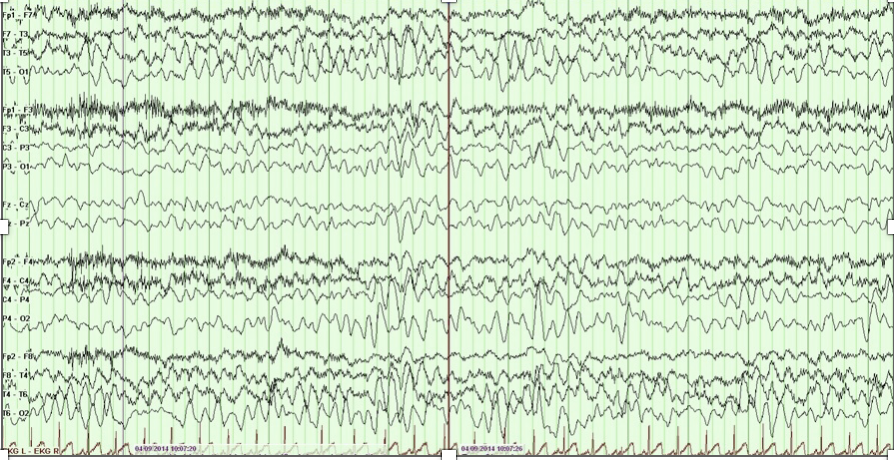


- 2 months (1)
- **10 months** (2)
- 5 years (3)
- 10 years (4)
- 16 years (5)

Q18 There is a 45-year-old woman who has the EEG noted below. What abnormality do you see in this EEG?


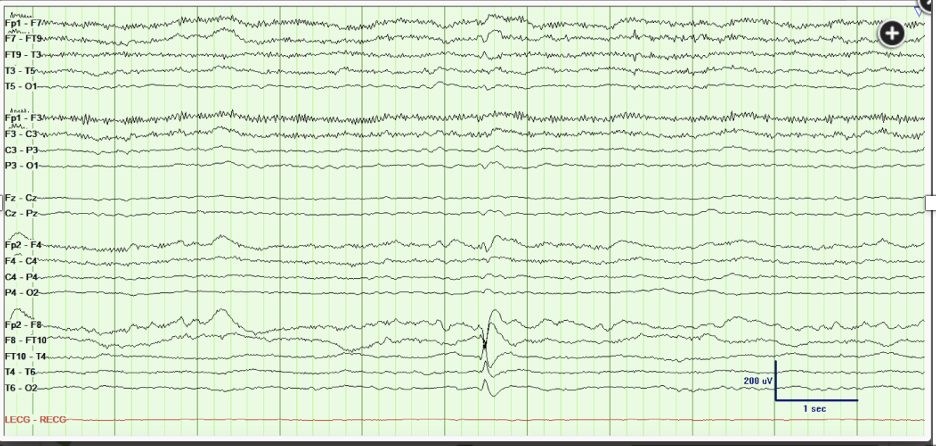


- **Temporal epileptiform discharge** (1)
- Occipital epileptiform discharge (2)
- Frontal epileptiform discharge (3)
- Central epileptiform discharge (4)
- Rolandic epileptiform discharge (5)

Q10 Which of the following is correct about posterior slow waves of youth?

- They consist of beta activity within the posterior dominant rhythm (1)
- They are abnormal in children (2)
- They are mainly seen during the sleep state in the occipital leads (3)
- **They consist of delta activity within the posterior dominant rhythm** (4)
- They block with eye closure (5)

Q14 What direction is the patient looking within the area highlighted by the box?


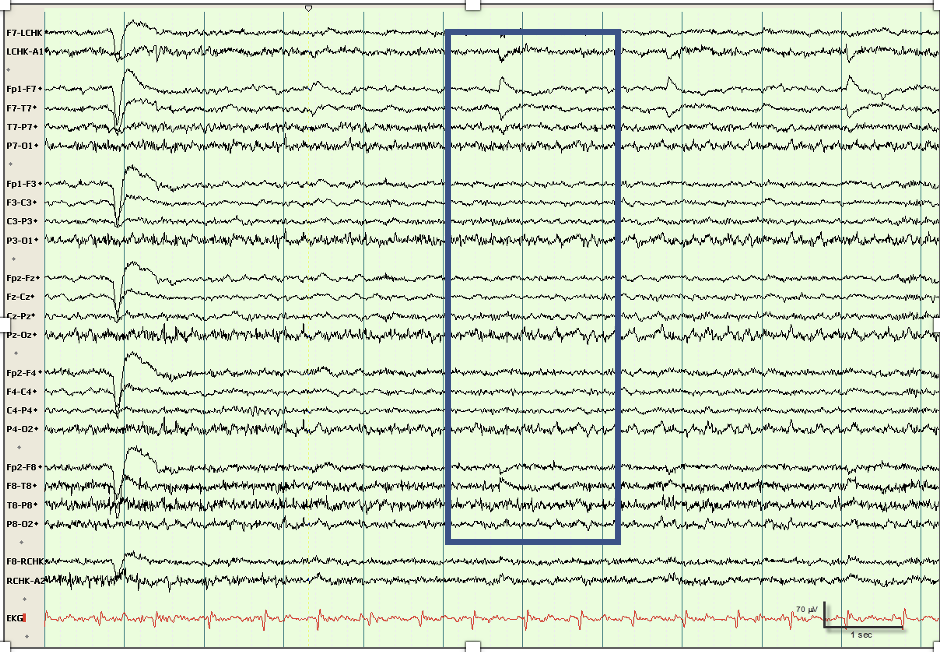


- Right (1)
- Up (2)
- **Left** (3)
- Down (4)

Q11 Which of the following is a component of N1 sleep?

- **POSTs** (1)
- Sleep spindles (2)
- K-complexes (3)
- High-voltage polymorphic delta (4)

Q12 The finding below appears first in which stage of sleep?


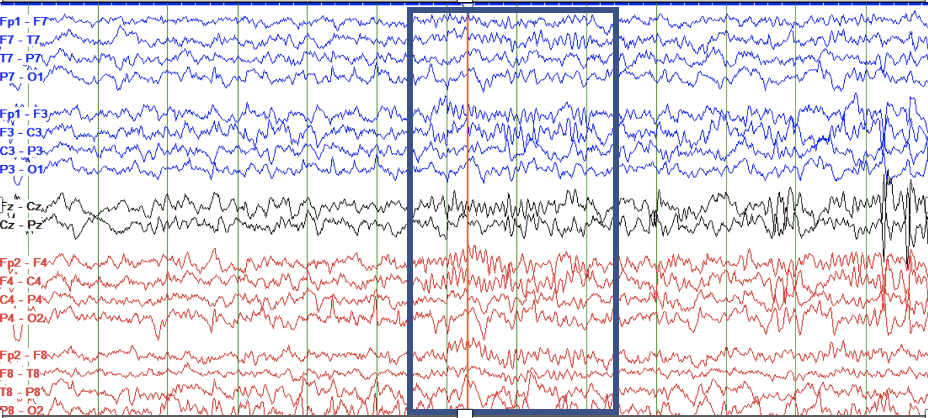


- N1 Sleep (1)
- **N2 Sleep** (2)
- N3 Sleep (3)
- REM Sleep (4)

Q16 A 30-year-old man with a known focal cortical dysplasia (FCD) is admitted to the Neuro- ICU confused, with very frequent clusters of rhythmic jerking of his left face and arm. He is intubated and sedated. See EEG image attached. How would you best classify this status presentation according to the ILAE Classification System for Status Epilepticus?


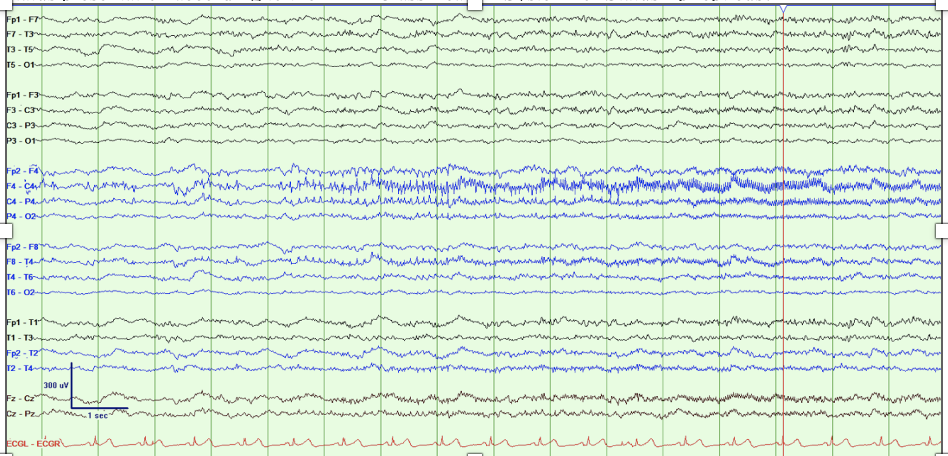


- Convulsive status epilepticus (1)
- Non-convulsive status epilepticus (2)
- **Focal motor status epilepticus with impaired consciousness** (3)
- Focal non-motor status epilepticus with impaired consciousness (4)
- Focal motor status epilepticus with secondary generalized convulsive status epilepticus (5)

Q13 At what age do sleep spindles first appear?

- Birth (1)
- **2 months of age** (2)
- 2 years of age (3)
- 4 years of age (4)
- 10 years of age (5)

Q17 What is a “breach rhythm”?

- A focal increase in the amplitude of low-frequency activity. (1)
- A focal decrease in the amplitude of low-frequency activity (2)
- **A focal increase in the amplitude of high-frequency activity.** (3)
- A focal decrease in the amplitude of high-frequency activity. (4)

Q19 What does the presence of the following pattern below suggest?


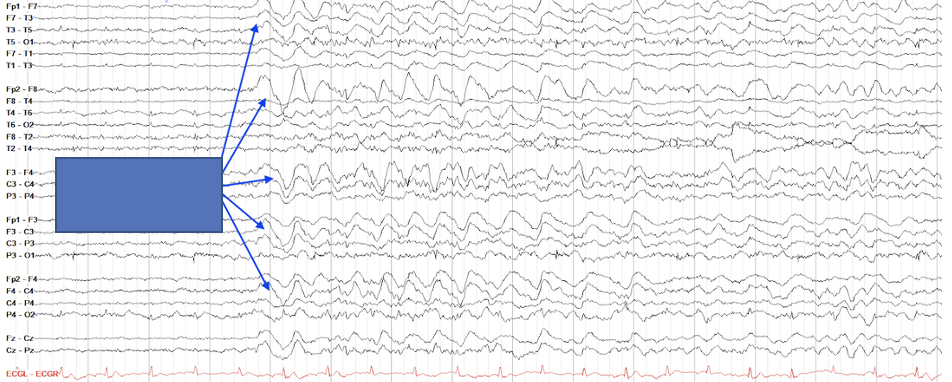


- Increased risk for seizures (1)
- **Nonspecific cerebral dysfunction** (2)
- Nothing: this is artifact from frequent eye blinks (3)
- This pattern is concerning for status epilepticus (4)

Q6 What is the name of the rhythm highlighted in the box below?

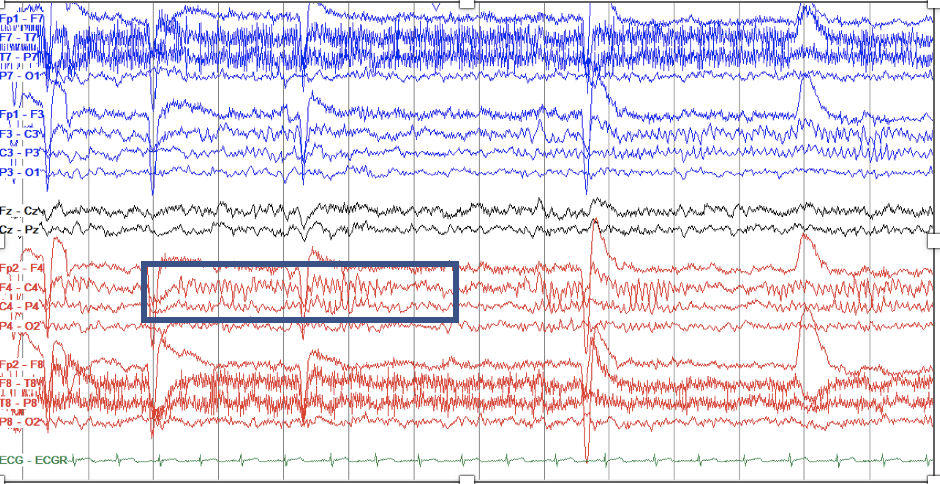


- Alpha rhythm (1)
- Third rhythm (2)
- **Mu rhythm** (3)
- Posterior dominant rhythm (4)
- Breech rhythm (5)

Q20 You are treating a 20-year-old woman whose EEG shows the findings below. What is her most likely diagnosis?


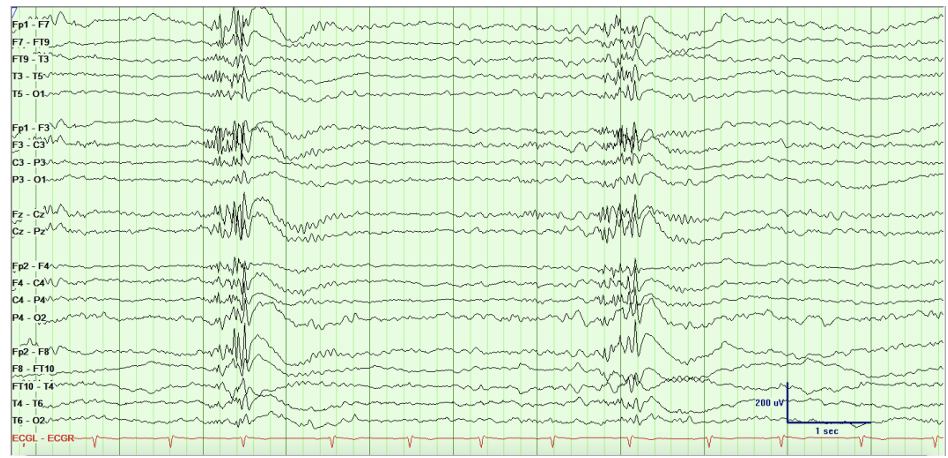


- **Juvenile myoclonic epilepsy** (1)
- Childhood absence epilepsy (2)
- Lennox-gastaut syndrome (3)
- West syndrome (4)
- Tonic epilepsy (5)

|  |
| --- |

Q21 Which of the following lists the typical settings in Natus for low frequency filter, high frequency filter, notch and sensitivity?

- 0.1, 80, 60, 7 (1)
- **1, 70, 60, 7** (2)
- 0.5, 80, 70, 10 (3)
- 2, 70, 60, 5 (4)

End of Block: The next questions will assess your current ability to read EEGs.

Start of Block: Block 2

Q44 The next questions will assess your current comfort with EEG reading

Q5 How confident are you that you can accurately read an EEG independently?

- Extremely confident (1)
- Very confident (2)
- Somewhat confident (3)
- Not so confident (4)
- Not at all confident (5)

Q6 How confident do you feel using EEG reading software (Natus)?

- Extremely confident (1)
- Very confident (2)
- Somewhat confident (3)
- Not so confident (4)
- Not at all confident (5)

Q7 What is your confidence level in identifying when you need help interpreting an EEG from an epilepsy trained neurologist?

- Extremely confident (1)
- Very confident (2)
- Somewhat confident (3)
- Not so confident (4)
- Not at all confident (5)

Q8 What is your confidence level in identifying patients for whom EEG is appropriate?

- Extremely confident (1)
- Very confident (2)
- Somewhat confident (3)
- Not so confident (4)
- Not at all confident (5)

Q9  Are you able to recognize normal EEG features of wake and sleep states in adults?

- I always recognize these features independently without help from a supervisor (1)
- I often recognize these features independently (2)
- I recognize these features but need help from a supervisor (3)
- I cannot recognize these features consistently (4)

Q10  Are you able to recognize normal EEG features of wake and sleep states in children?

- I always recognize these features independently without help from a supervisor (1)
- I often recognize these features independently (2)
- I recognize these features but need help from a supervisor (3)
- I cannot recognize these features consistently (4)

Q11 Are you able to recognize common EEG artifacts in adults?

- I always recognize these features independently without help from a supervisor (1)
- I often recognize these features independently (2)
- I recognize these features but need help from a supervisor (3)
- I cannot recognize these features consistently (4)

Q12 Are you able to recognize common EEG artifacts in children?

- I always recognize these features independently without help from a supervisor (1)
- I often recognize these features independently (2)
- I recognize these features but need help from a supervisor (3)
- I cannot recognize these features consistently (4)

Q13 Are you able to recognize EEG patterns of status epilepticus in adults?

- I always recognize these features independently without help from a supervisor (1)
- I often recognize these features independently (2)
- I recognize these features but need help from a supervisor (3)
- I cannot recognize these features consistently (4)

Q14 Are you able to recognize EEG patterns of status epilepticus in children?

- I always recognize these features independently without help from a supervisor (1)
- I often recognize these features independently (2)
- I recognize these features but need help from a supervisor (3)
- I cannot recognize these features consistently (4)

Q15 Are you able to recognize common EEG abnormalities in adults?

- I always recognize these features independently without help from a supervisor (1)
- I often recognize these features independently (2)
- I recognize these features but need help from a supervisor (3)
- I cannot recognize these features consistently (4)

Q16 Are you able to recognize common EEG abnormalities in children?

- I always recognize these features independently without help from a supervisor (1)
- I often recognize these features independently (2)
- I recognize these features but need help from a supervisor (3)
- I cannot recognize these features consistently (4)

Q17 Are you able to recognize normal EEG variants in adults?

- I always recognize these features independently without help from a supervisor (1)
- I often recognize these features independently (2)
- I recognize these features but need help from a supervisor (3)
- I cannot recognize these features consistently (4)

Q18 Are you able to recognize normal EEG variants in children?

- I always recognize these features independently without help from a supervisor (1)
- I often recognize these features independently (2)
- I recognize these features but need help from a supervisor (3)
- I cannot recognize these features consistently (4)

Q19  Can you differentiate abnormalities in the ictal-interictal continuum from patterns that could represent status epilepticus in adults?

- I always recognize these features independently without help from a supervisor (1)
- I often recognize these features independently (2)
- I recognize these features but need help from a supervisor (3)
- I cannot recognize these features consistently (4)

Q20  Can you differentiate abnormalities in the ictal-interictal continuum from patterns that could represent status epilepticus in children?

- I always recognize these features independently without help from a supervisor (1)
- I often recognize these features independently (2)
- I recognize these features but need help from a supervisor (3)
- I cannot recognize these features consistently (4)

Q21 Can you interpret uncommon EEG abnormalities in adults?

- I always recognize these features independently without help from a supervisor (1)
- I often recognize these features independently (2)
- I recognize these features but need help from a supervisor (3)
- I cannot recognize these features consistently (4)

Q22 Can you interpret uncommon EEG abnormalities in children?

- I always recognize these features independently without help from a supervisor (1)
- I often recognize these features independently (2)
- I recognize these features but need help from a supervisor (3)
- I cannot recognize these features consistently (4)

Q23 Can you independently write an EEG report?

- I always independently generate a report without help from a supervisor (1)
- I am often able to generate a report independently (2)
- I need help from a supervisor to generate a report (3)
- I cannot generate a report independently (4)

Q26 How many EEGs do you estimate you have read during residency? "Read" means reviewed an EEG in its entirety, but does not need to include drafting an EEG report.

________________________________________________________________

Q53 How many EEGs do you estimate you have read during residency? "Read" means reviewed an EEG in its entirety, but does not need to include drafting an EEG report.

________________________________________________________________

Q27 How many EEG reports do you estimate you have written during residency?

________________________________________________________________

| Page Break |  |
| --- | --- |

Q46 Please tell us a little bit about yourself.

Q35 What is your current gender identify? Please select all that apply.

- Male/Man (1)
- Female/Woman (2)
- Non-binary (3)
- Prefer not to say (4)
- Other (please specify) (5) __________________________________________________

Q36 What is your ethnic/racial identity? Please select all that apply.

- Black/African American (1)
- Latinx or Hispanic (2)
- Asian (3)
- Pacific Islander or Native Hawaiian (4)
- White/Caucasian (5)
- Indigenous/Native American (6)
- Middle Eastern/North African (7)
- Prefer not to say (8)
- Other (please specify) (9) __________________________________________________

Q37  Are you an adult neurology or child neurology resident?

- Adult neurology (1)
- Child neurology (2)
- Other (please specify) (3) __________________________________________________

Q38 What is your level of training?

- PGY-1 (1)
- PGY-2 (2)
- PGY-3 (3)
- PGY-4 (4)
- PGY-5 (5)
- Other (please specify) (6)

Q39 What area/subspecialty of neurology do you plan to practice in for your future career (i.e. general outpatient neurology, neurohospitalist, neuromuscular, epilepsy etc.). ?

________________________________________________________________

End of Block: Block 2

Post-Survey EEG Education

Start of Block: Default Question Block

Q1  Please enter your full name. This information will be used only to compare pre- and post-survey results and will be re-coded for anonymity prior to analysis.

________________________________________________________________

| Page Break |  |
| --- | --- |

Q31 The next questions will assess your ability to read EEGs.

Q2 A 33-year-old woman presents to the emergency room after having a seizure. Your workup includes a routine EEG prior to the patient leaving the emergency room. Which of the following statements is true?

- **The presence of focal interictal epileptiform discharges on EEG has a high positive predictive value for epilepsy** (1)
- The absence of focal interictal epileptiform discharges on EEG will rule out epilepsy (2)
- If the event concerning for seizure is not captured on EEG, the test will not help predict the patient's risk of epilepsy (3)
- The frequency of focal interictal epileptiform discharges on EEG does not have significant prognostic value (4)

Q3 When is the rhythm outlined in the box below best seen on EEG?

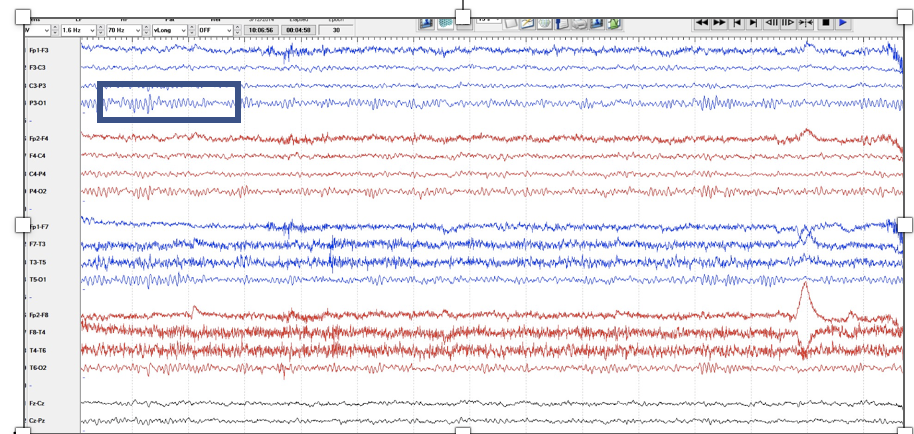


- During drowsiness (1)
- During thoughts of moving the contralateral arm (2)
- **During wakefulness with eyes closed** (3)
- During strenuous mental tasks (4)
- During sleep (5)

Q5 What is the patient most likely doing during the EEG segment below?


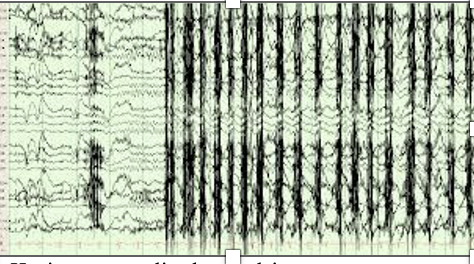


- Having a generalized convulsion (1)
- Walking around the room (2)
- **Eating a snack** (3)
- Shaking their head from side to side (4)

Q15 What is the likelihood that this EEG pattern is consistent with non-convulsive status epilepticus (NCSE) in a patient with no clinical correlate to this pattern?

  
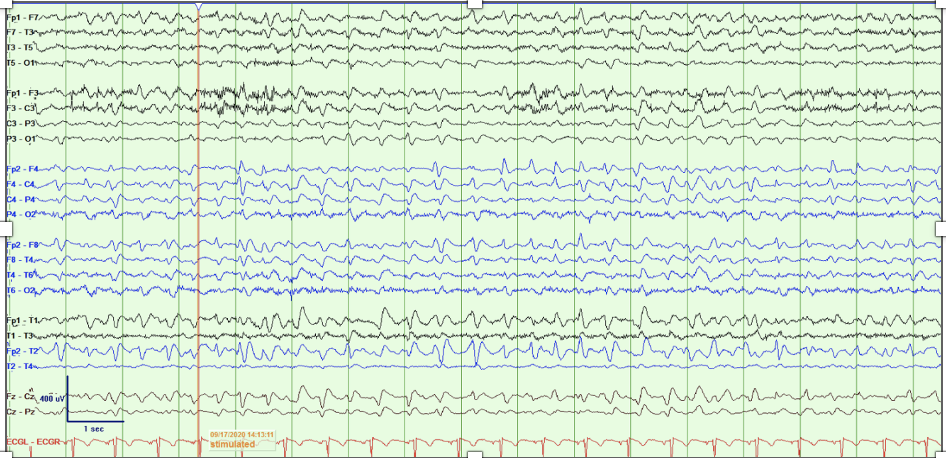


- **The EEG is probably not consistent with NCSE because the rhythmic discharges are slower than 2.5hz and do not evolve** (1)
- This EEG is probably not consistent with NCSE because the rhythmic discharges are triphasic appearing (2)
- This EEG is probably NCSE because there are rhythmic discharges greater than 2.5hz (3)
- This EEG is probably NCSE because this pattern emerged after stimulation of the patient (4)

Q8 In the displayed EEG, what is the box over the right temporal region highlighting?


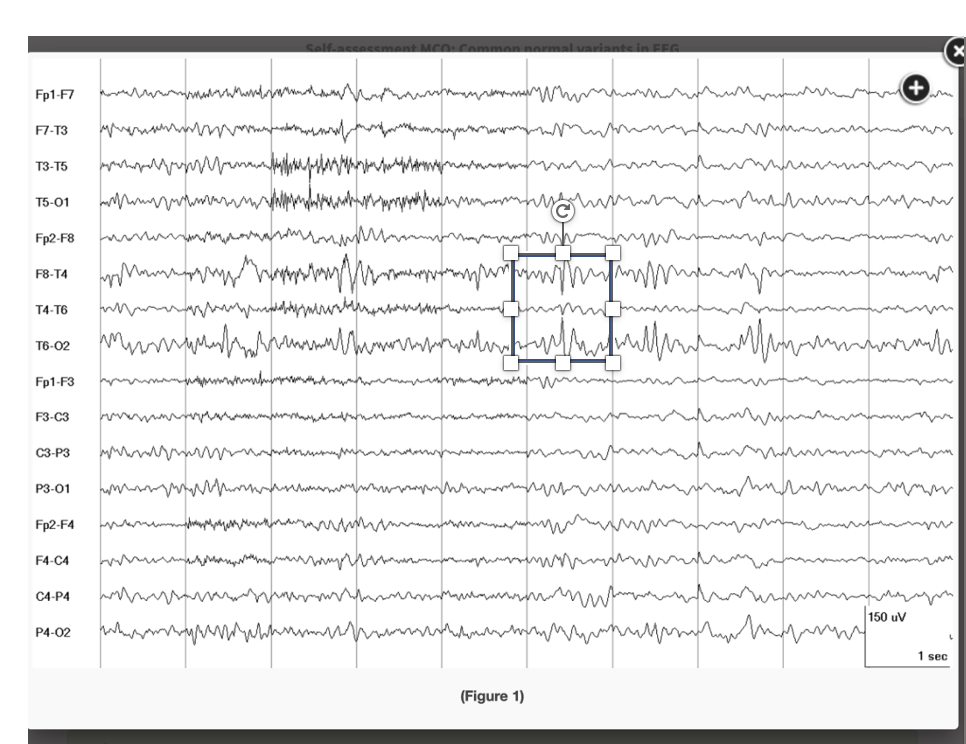


- Mu rhythm (1)
- **Wicket Spike** (2)
- Temporal Spike (3)
- POSTs (4)

Q9 How old is the patient whose normal awake EEG is pictured here?


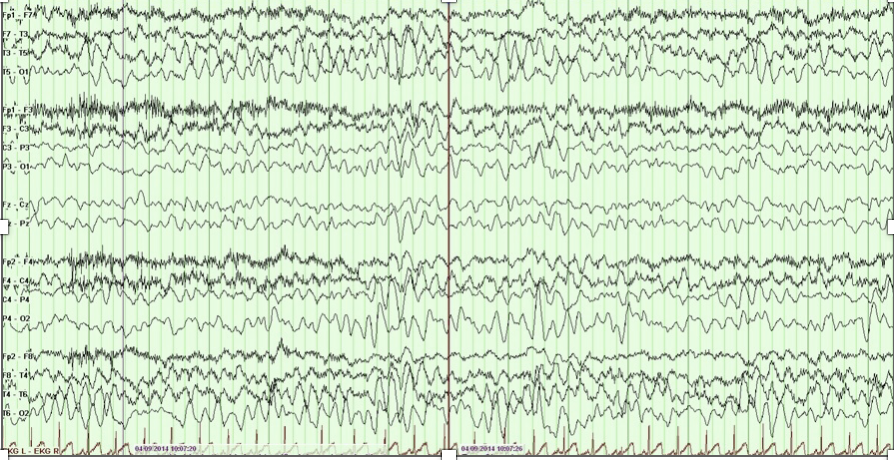


- 2 months (1)
- **10 months (**2)
- 5 years (3)
- 10 years (4)
- 16 years (5)

Q18 There is a 45-year-old woman who has the EEG noted below. What abnormality do you see in this EEG?


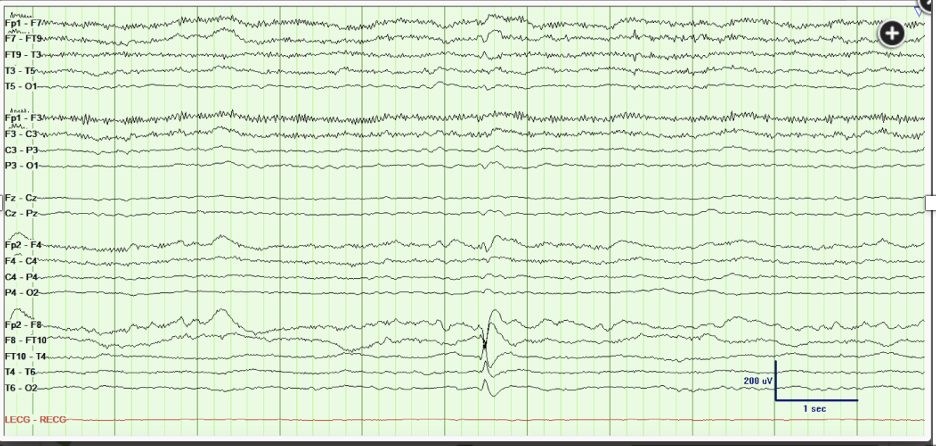


- **Temporal epileptiform discharge** (1)
- Occipital epileptiform discharge (2)
- Frontal epileptiform discharge (3)
- Central epileptiform discharge (4)
- Rolandic epileptiform discharge (5)

Q10 Which of the following is correct about posterior slow waves of youth?

- They consist of beta activity within the posterior dominant rhythm (1)
- They are abnormal in children (2)
- They are mainly seen during the sleep state in the occipital leads (3)
- **They consist of delta activity within the posterior dominant rhythm** (4)
- They block with eye closure (5)

Q14 What direction is the patient looking within the area highlighted by the box?


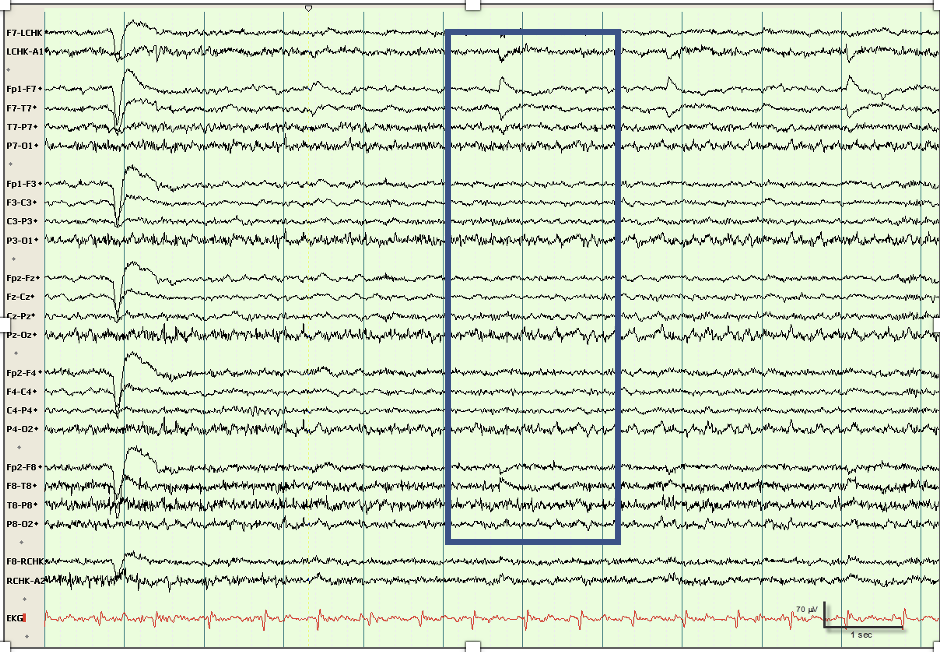


- Right (1)
- Up (2)
- **Left** (3)
- Down (4)

Q11 Which of the following is a component of N1 sleep?

- **POSTs** (1)
- Sleep spindles (2)
- K-complexes (3)
- High-voltage polymorphic delta (4)

Q12 The finding below appears first in which stage of sleep?


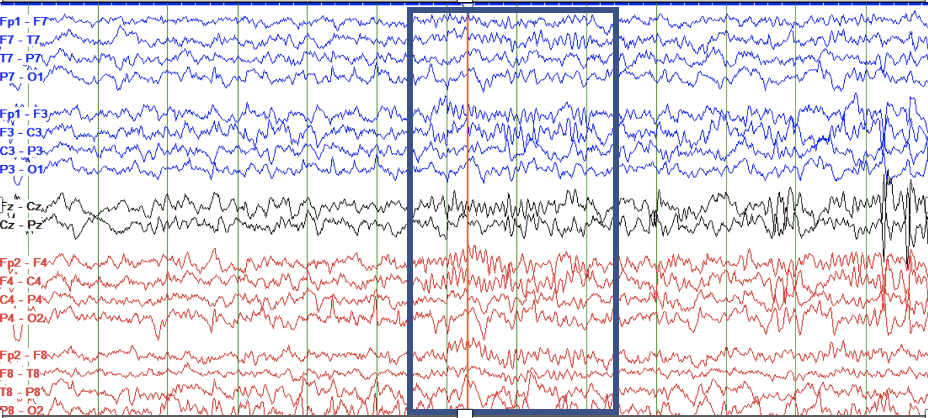


- N1 Sleep (1)
- **N2 Sleep** (2)
- N3 Sleep (3)
- REM Sleep (4)

Q16 A 30-year-old man with a known focal cortical dysplasia (FCD) is admitted to the Neuro- ICU confused, with very frequent clusters of rhythmic jerking of his left face and arm. He is intubated and sedated. See EEG image attached. How would you best classify this status presentation according to the ILAE Classification System for Status Epilepticus?


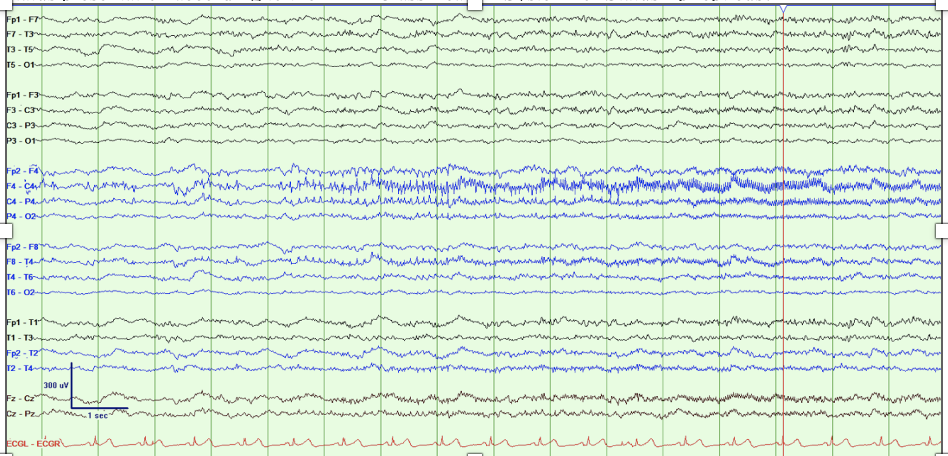


- Convulsive status epilepticus (1)
- Non-convulsive status epilepticus (2)
- **Focal motor status epilepticus with impaired consciousness** (3)
- Focal non-motor status epilepticus with impaired consciousness (4)
- Focal motor status epilepticus with secondary generalized convulsive status epilepticus (5)

Q13 At what age do sleep spindles first appear?

- Birth (1)
- **2 months of age** (2)
- 2 years of age (3)
- 4 years of age (4)
- 10 years of age (5)

Q17 What is a “breach rhythm”?

- A focal increase in the amplitude of low-frequency activity. (1)
- A focal decrease in the amplitude of low-frequency activity (2)
- **A focal increase in the amplitude of high-frequency activity**. (3)
- A focal decrease in the amplitude of high-frequency activity. (4)

Q19 What does the presence of the following pattern below suggest?


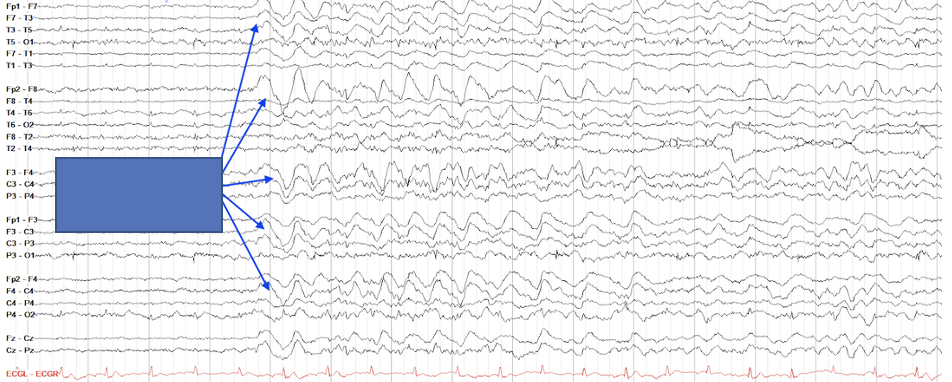


- Increased risk for seizures (1)
- **Nonspecific cerebral dysfunction** (2)
- Nothing: this is artifact from frequent eye blinks (3)
- This pattern is concerning for status epilepticus (4)

Q6 What is the name of the rhythm highlighted in the box below?

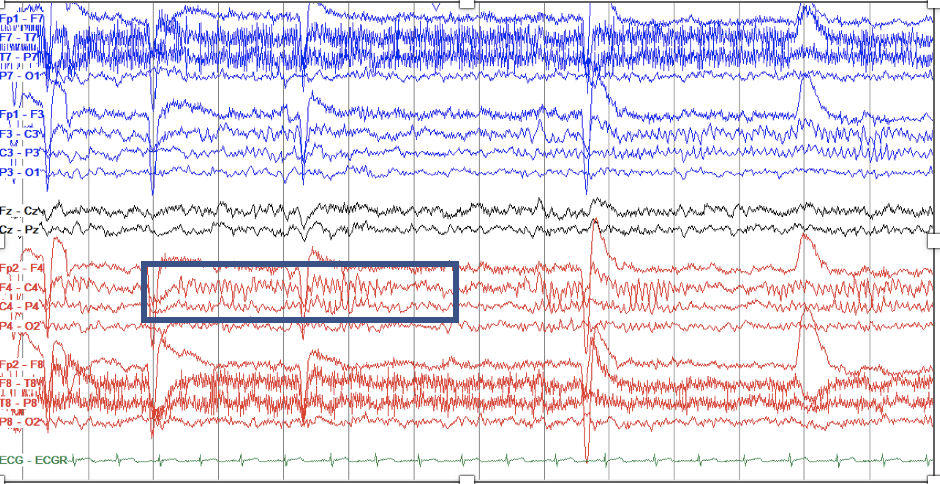


- Alpha rhythm (1)
- Third rhythm (2)
- **Mu rhythm** (3)
- Posterior dominant rhythm (4)
- Breech rhythm (5)

Q20 You are treating a 20-year-old woman whose EEG shows the findings below. What is her most likely diagnosis?


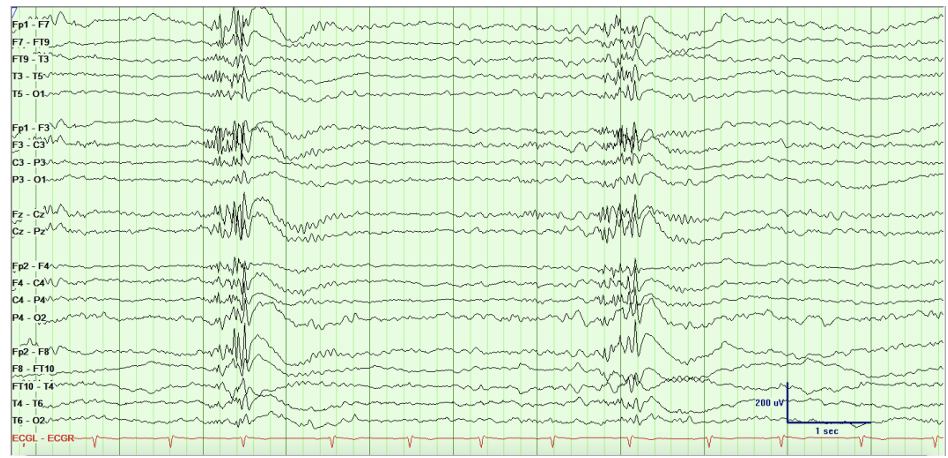


- **Juvenile myoclonic epilepsy** (1)
- Childhood absence epilepsy (2)
- Lennox-gastaut syndrome (3)
- West syndrome (4)
- Tonic epilepsy (5)

|  |
| --- |

Q21 Which of the following lists the typical settings in Natus for low frequency filter, high frequency filter, notch and sensitivity?

- 0.1, 80, 60, 7 (1)
- **1, 70, 60, 7** (2)
- 0.5, 80, 70, 10 (3)
- 2, 70, 60, 5 (4)

| Page Break |  |
| --- | --- |

Q44 The next questions will assess your current comfort with EEG reading

Q5 How confident are you that you can accurately read an EEG independently?

- Extremely confident
- Very confident
- Somewhat confident
- Not so confident
- Not at all confident

Q6 How confident do you feel using EEG reading software (Natus)?

- Extremely confident
- Very confident
- Somewhat confident
- Not so confident
- Not at all confident

Q7 What is your confidence level in identifying when you need help interpreting an EEG from an epilepsy trained neurologist?

- Extremely confident
- Very confident
- Somewhat confident
- Not so confident
- Not at all confident

Q8 What is your confidence level in identifying patients for whom EEG is appropriate?

- Extremely confident
- Very confident
- Somewhat confident
- Not so confident
- Not at all confident

Q9  Are you able to recognize normal EEG features of wake and sleep states in adults?

- I always recognize these features independently without help from a supervisor
- I often recognize these features independently
- I recognize these features but need help from a supervisor
- I cannot recognize these features consistently

Q10  Are you able to recognize normal EEG features of wake and sleep states in children?

- I always recognize these features independently without help from a supervisor
- I often recognize these features independently
- I recognize these features but need help from a supervisor
- I cannot recognize these features consistently

Q11 Are you able to recognize common EEG artifacts in adults?

- I always recognize these features independently without help from a supervisor
- I often recognize these features independently
- I recognize these features but need help from a supervisor
- I cannot recognize these features consistently

Q12 Are you able to recognize common EEG artifacts in children?

- I always recognize these features independently without help from a supervisor
- I often recognize these features independently
- I recognize these features but need help from a supervisor
- I cannot recognize these features consistently

Q13 Are you able to recognize EEG patterns of status epilepticus in adults?

- I always recognize these features independently without help from a supervisor
- I often recognize these features independently
- I recognize these features but need help from a supervisor
- I cannot recognize these features consistently

Q14 Are you able to recognize EEG patterns of status epilepticus in children?

- I always recognize these features independently without help from a supervisor
- I often recognize these features independently
- I recognize these features but need help from a supervisor
- I cannot recognize these features consistently

Q15 Are you able to recognize common EEG abnormalities in adults?

- I always recognize these features independently without help from a supervisor
- I often recognize these features independently
- I recognize these features but need help from a supervisor
- I cannot recognize these features consistently

Q16 Are you able to recognize common EEG abnormalities in children?

- I always recognize these features independently without help from a supervisor
- I often recognize these features independently
- I recognize these features but need help from a supervisor
- I cannot recognize these features consistently

Q17 Are you able to recognize normal EEG variants in adults?

- I always recognize these features independently without help from a supervisor
- I often recognize these features independently
- I recognize these features but need help from a supervisor
- I cannot recognize these features consistently

Q18 Are you able to recognize normal EEG variants in children?

- I always recognize these features independently without help from a supervisor
- I often recognize these features independently
- I recognize these features but need help from a supervisor
- I cannot recognize these features consistently

Q19  Can you differentiate abnormalities in the ictal-interictal continuum from patterns that could represent status epilepticus in adults?

- I always recognize these features independently without help from a supervisor
- I often recognize these features independently
- I recognize these features but need help from a supervisor
- I cannot recognize these features consistently

Q20  Can you differentiate abnormalities in the ictal-interictal continuum from patterns that could represent status epilepticus in children?

- I always recognize these features independently without help from a supervisor
- I often recognize these features independently
- I recognize these features but need help from a supervisor
- I cannot recognize these features consistently

Q21 Can you interpret uncommon EEG abnormalities in adults?

- I always recognize these features independently without help from a supervisor
- I often recognize these features independently
- I recognize these features but need help from a supervisor
- I cannot recognize these features consistently

Q22 Can you interpret uncommon EEG abnormalities in children?

- I always recognize these features independently without help from a supervisor
- I often recognize these features independently
- I recognize these features but need help from a supervisor
- I cannot recognize these features consistently

Q23 Can you independently write an EEG report?

- I always independently generate a report without help from a supervisor
- I am often able to generate a report independently
- I need help from a supervisor to generate a report
- I cannot generate a report independently

End of Block: Default Question Block

Start of Block: Block 1

Q23 The next questions will assess your experience with the EEG education on your pediatric epilepsy or EEG reading rotation.

Q25 During which rotation did you complete the educational materials?

- Pediatric epilepsy
- EEG reading rotation
- Other (Please specify) __________________________________________________

Q26 How many EEGs did you review during this rotation?

________________________________________________________________

Q27 How many EEG reports did you write during this rotation?

________________________________________________________________

Q28 Please indicate the efficacy of the educational materials you used.

|  | Did Not Use | Unhelpful | Slightly Helpful | Helpful | Very Helpful |
| --- | --- | --- | --- | --- | --- |
| Normal EEG PowerPoint |  |  |  |  |  |
| Abnormal EEG PowerPoint |  |  |  |  |  |
| Using Natus PowerPoint |  |  |  |  |  |
| Learningeeg.com |  |  |  |  |  |
| Independent EEG reading (not staffed) |  |  |  |  |  |
| EEG reading followed by staffing with attending or fellow |  |  |  |  |  |
| Writing EEG reports |  |  |  |  |  |
| EEG atlas creation |  |  |  |  |  |

Q29 Which parts of the education did you find most helpful?

________________________________________________________________

Q30 What can we do to improve future education experiences?

________________________________________________________________

End of Block: Block 1

3-Month Post Survey

Start of Block: Default Question Block

Q1  Please enter your full name. This information will be used only to compare pre- and post-survey results and will be re-coded for anonymity prior to analysis.

________________________________________________________________

| Page Break |  |
| --- | --- |

Q31 The next questions will assess your ability to read EEGs.

Q2 A 33-year-old woman presents to the emergency room after having a seizure. Your workup includes a routine EEG prior to the patient leaving the emergency room. Which of the following statements is true?

- **The presence of focal interictal epileptiform discharges on EEG has a high positive predictive value for epilepsy** (1)
- The absence of focal interictal epileptiform discharges on EEG will rule out epilepsy (2)
- If the event concerning for seizure is not captured on EEG, the test will not help predict the patient's risk of epilepsy (3)
- The frequency of focal interictal epileptiform discharges on EEG does not have significant prognostic value (4)

Q3 When is the rhythm outlined in the box below best seen on EEG?


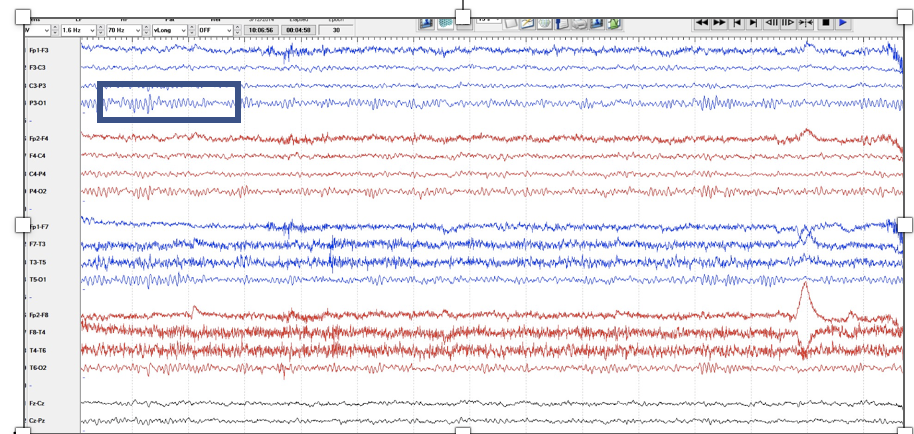


- During drowsiness (1)
- During thoughts of moving the contralateral arm (2)
- **During wakefulness with eyes closed** (3)
- During strenuous mental tasks (4)
- During sleep (5)

Q5 What is the patient most likely doing during the EEG segment below?


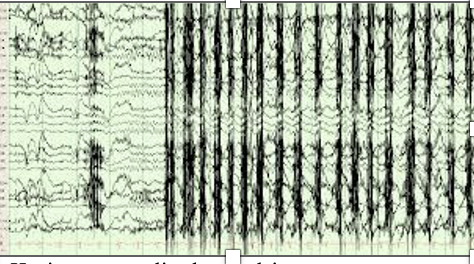


- Having a generalized convulsion (1)
- Walking around the room (2)
- **Eating a snack** (3)
- Shaking their head from side to side (4)

Q15 What is the likelihood that this EEG pattern is consistent with non-convulsive status epilepticus (NCSE) in a patient with no clinical correlate to this pattern?

  
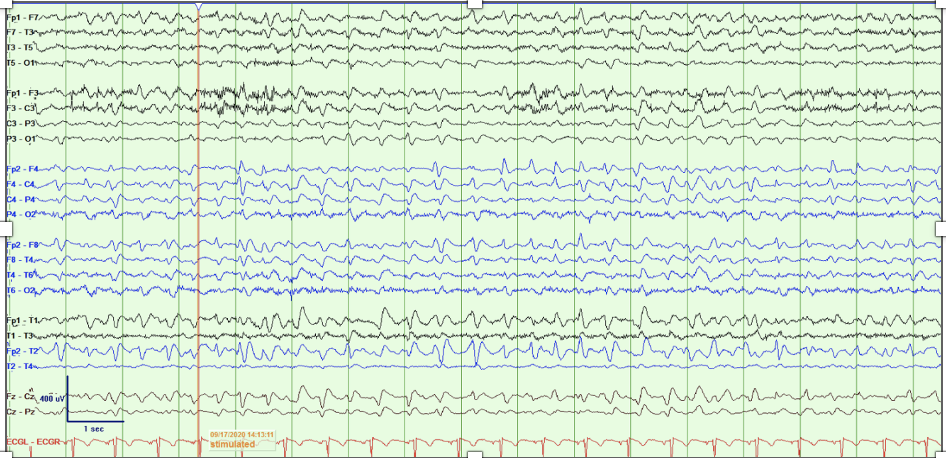


- **The EEG is probably not consistent with NCSE because the rhythmic discharges are slower than 2.5hz and do not evolve** (1)
- This EEG is probably not consistent with NCSE because the rhythmic discharges are triphasic appearing (2)
- This EEG is probably NCSE because there are rhythmic discharges greater than 2.5hz (3)
- This EEG is probably NCSE because this pattern emerged after stimulation of the patient (4)

Q8 In the displayed EEG, what is the box over the right temporal region highlighting?


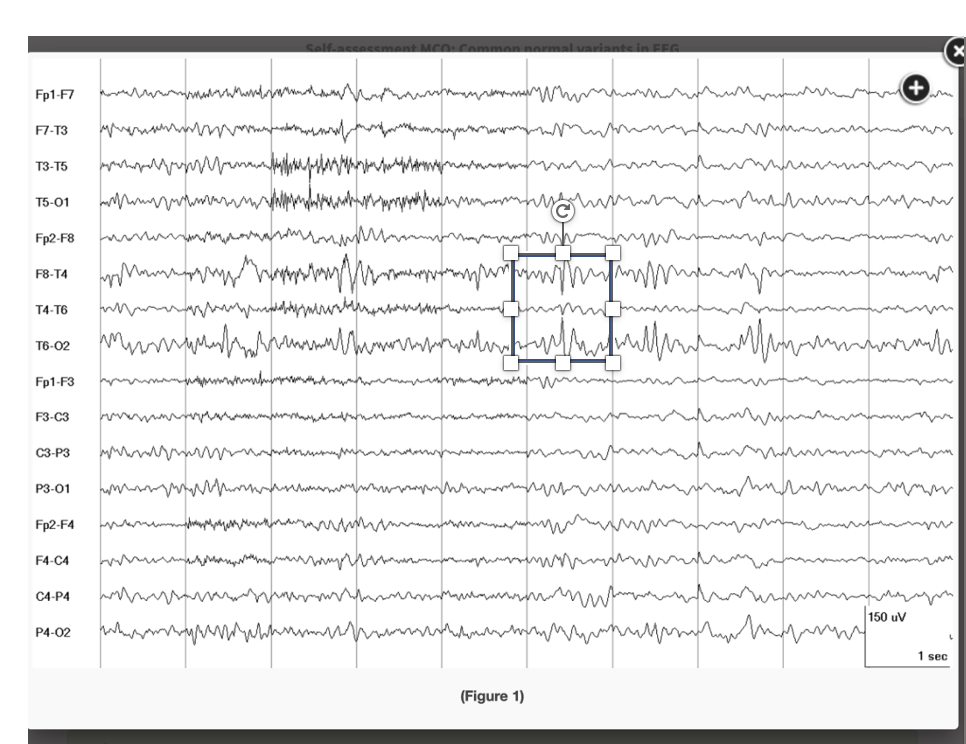


- Mu rhythm (1)
- **Wicket Spike** (2)
- Temporal Spike (3)
- POSTs (4)

Q9 How old is the patient whose normal awake EEG is pictured here?


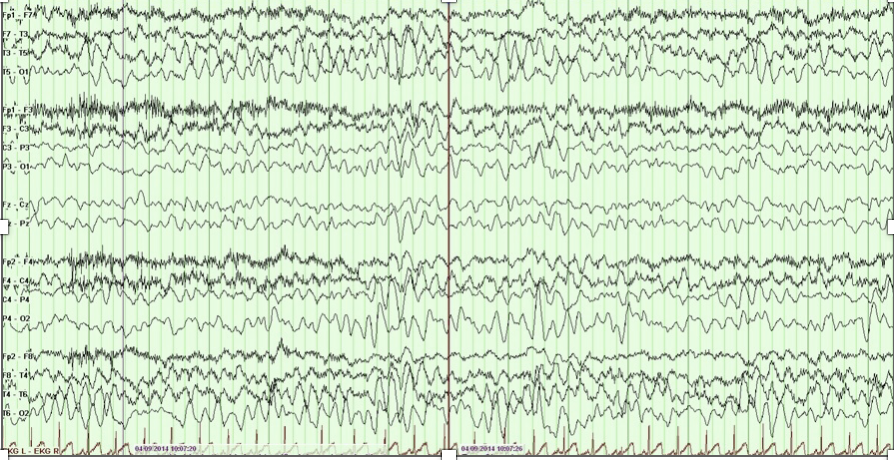


- 2 months (1)
- **10 months** (2)
- 5 years (3)
- 10 years (4)
- 16 years (5)

Q18 There is a 45-year-old woman who has the EEG noted below. What abnormality do you see in this EEG?


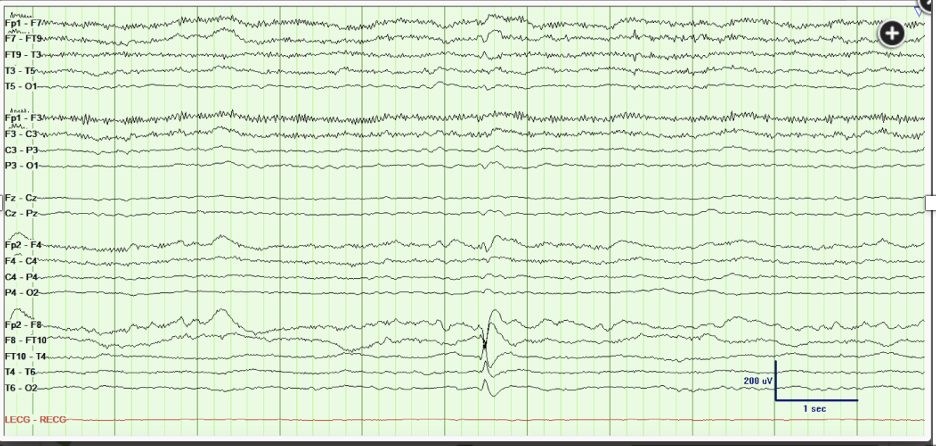


- **Temporal epileptiform discharge** (1)
- Occipital epileptiform discharge (2)
- Frontal epileptiform discharge (3)
- Central epileptiform discharge (4)
- Rolandic epileptiform discharge (5)

Q10 Which of the following is correct about posterior slow waves of youth?

- They consist of beta activity within the posterior dominant rhythm (1)
- They are abnormal in children (2)
- They are mainly seen during the sleep state in the occipital leads (3)
- **They consist of delta activity within the posterior dominant rhythm** (4)
- They block with eye closure (5)

Q14 What direction is the patient looking within the area highlighted by the box?


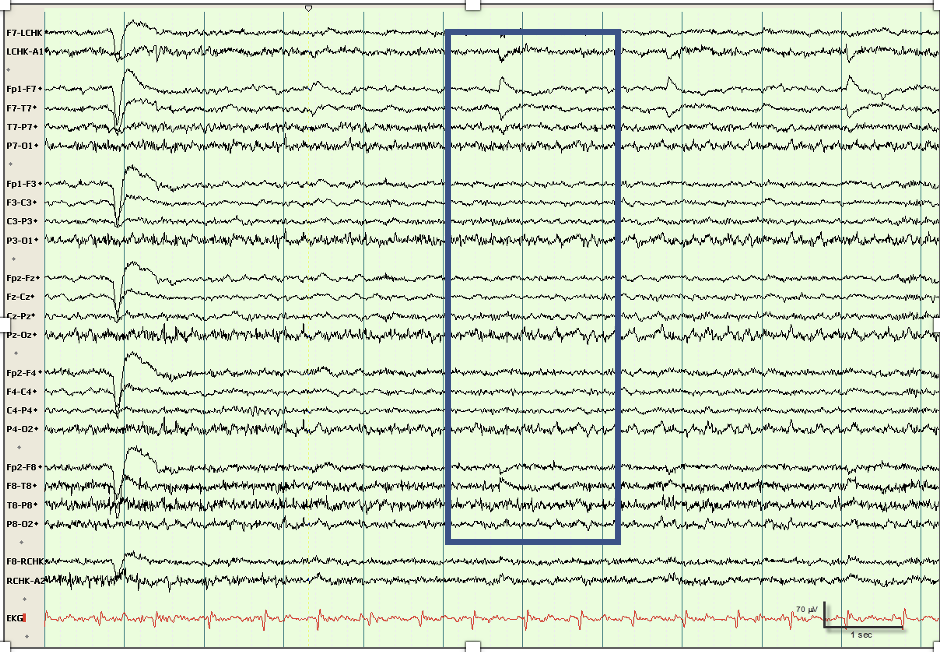


- Right (1)
- Up (2)
- **Left**  (3)
- Down (4)

Q11 Which of the following is a component of N1 sleep?

- **POSTs**  (1)
- Sleep spindles (2)
- K-complexes (3)
- High-voltage polymorphic delta (4)

Q12 The finding below appears first in which stage of sleep?


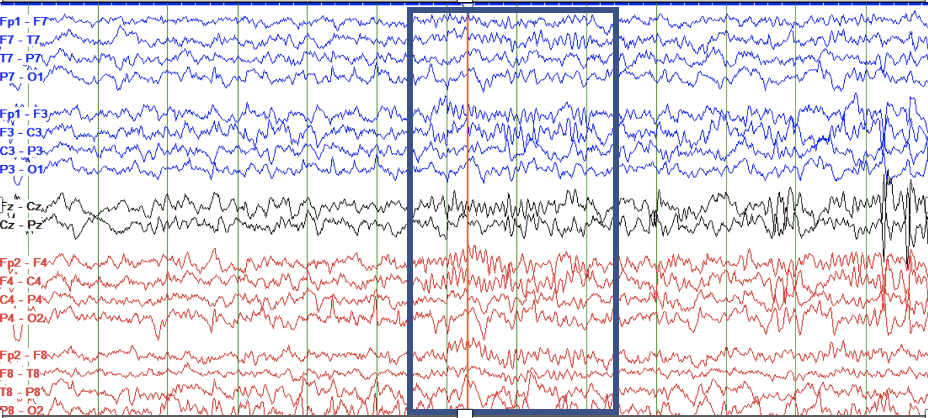


- N1 Sleep (1)
- **N2 Sleep** (2)
- N3 Sleep (3)
- REM Sleep (4)

Q16 A 30-year-old man with a known focal cortical dysplasia (FCD) is admitted to the Neuro- ICU confused, with very frequent clusters of rhythmic jerking of his left face and arm. He is intubated and sedated. See EEG image attached. How would you best classify this status presentation according to the ILAE Classification System for Status Epilepticus?


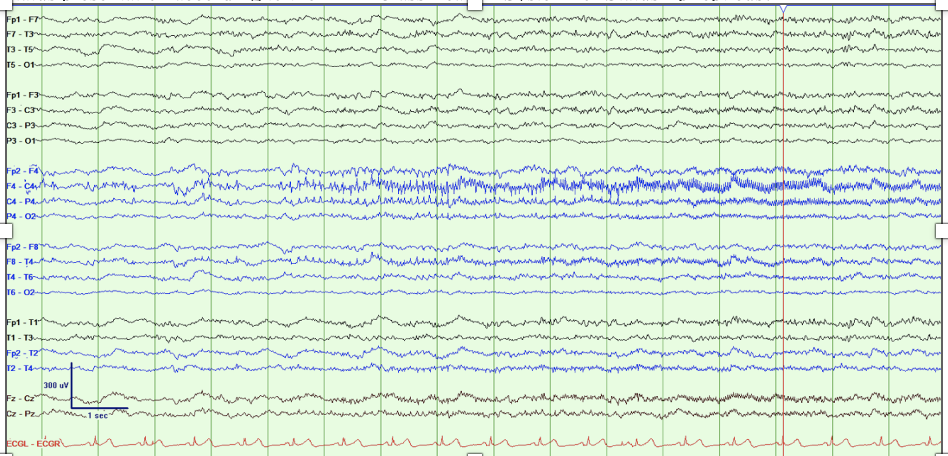


- Convulsive status epilepticus (1)
- Non-convulsive status epilepticus (2)
- **Focal motor status epilepticus with impaired consciousness** (3)
- Focal non-motor status epilepticus with impaired consciousness (4)
- Focal motor status epilepticus with secondary generalized convulsive status epilepticus (5)

Q13 At what age do sleep spindles first appear?

- Birth (1)
- **2 months of age** (2)
- 2 years of age (3)
- 4 years of age (4)
- 10 years of age (5)

Q17 What is a “breach rhythm”?

- A focal increase in the amplitude of low-frequency activity. (1)
- A focal decrease in the amplitude of low-frequency activity (2)
- **A focal increase in the amplitude of high-frequency activity**. (3)
- A focal decrease in the amplitude of high-frequency activity. (4)

Q19 What does the presence of the following pattern below suggest?


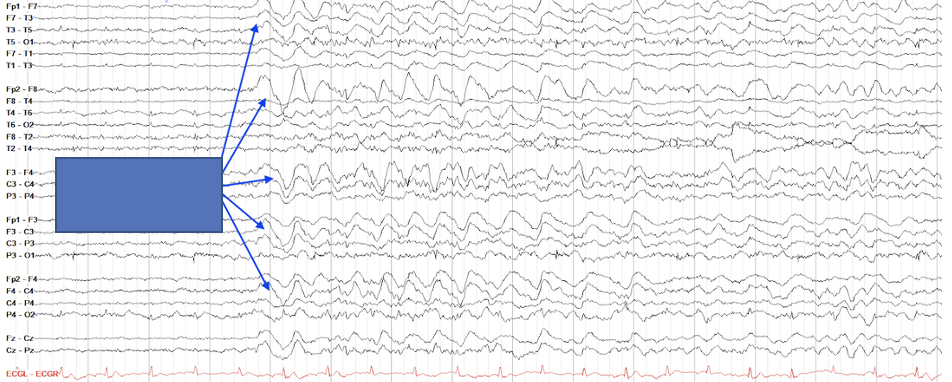


- Increased risk for seizures (1)
- **Nonspecific cerebral dysfunction** (2)
- Nothing: this is artifact from frequent eye blinks (3)
- This pattern is concerning for status epilepticus (4)

Q6 What is the name of the rhythm highlighted in the box below?

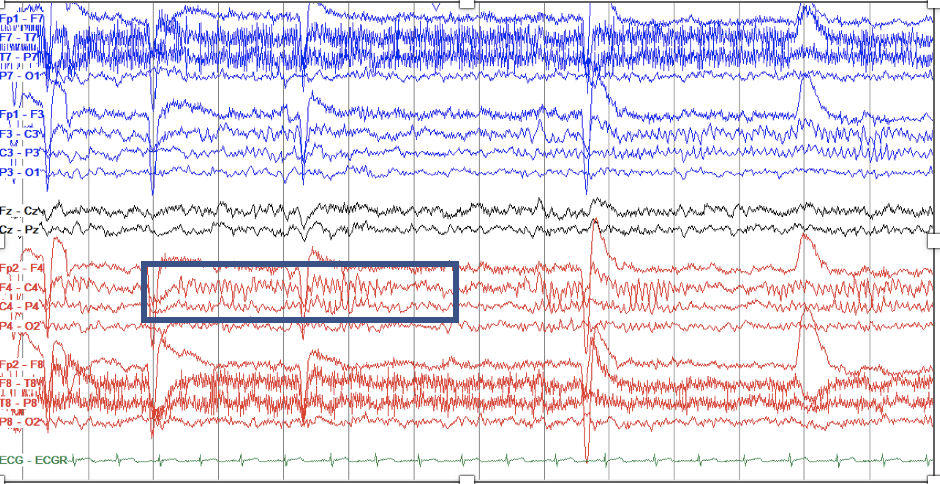


- Alpha rhythm (1)
- Third rhythm (2)
- **Mu rhythm** (3)
- Posterior dominant rhythm (4)
- Breech rhythm (5)

Q20 You are treating a 20-year-old woman whose EEG shows the findings below. What is her most likely diagnosis?


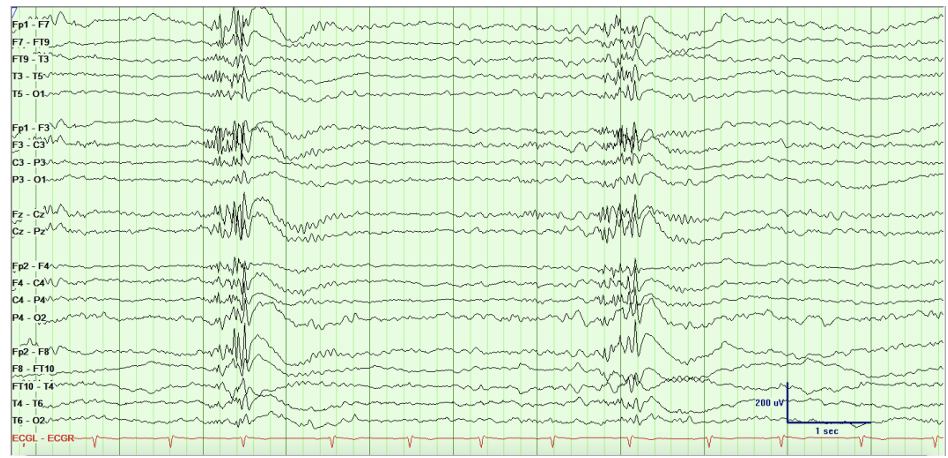


- **Juvenile myoclonic epilepsy** (1)
- Childhood absence epilepsy (2)
- Lennox-gastaut syndrome (3)
- West syndrome (4)
- Tonic epilepsy (5)

|  |
| --- |

Q21 Which of the following lists the typical settings in Natus for low frequency filter, high frequency filter, notch and sensitivity?

- 0.1, 80, 60, 7 (1)
- **1, 70, 60, 7** (2)
- 0.5, 80, 70, 10 (3)
- 2, 70, 60, 5 (4)

| Page Break |  |
| --- | --- |

Q44 The next questions will assess your current comfort with EEG reading

Q5 How confident are you that you can accurately read an EEG independently?

- Extremely confident
- Very confident
- Somewhat confident
- Not so confident
- Not at all confident

Q6 How confident do you feel using EEG reading software (Natus)?

- Extremely confident
- Very confident
- Somewhat confident
- Not so confident
- Not at all confident

Q7 What is your confidence level in identifying when you need help interpreting an EEG from an epilepsy trained neurologist?

- Extremely confident
- Very confident
- Somewhat confident
- Not so confident
- Not at all confident

Q8 What is your confidence level in identifying patients for whom EEG is appropriate?

- Extremely confident
- Very confident
- Somewhat confident
- Not so confident
- Not at all confident

Q9  Are you able to recognize normal EEG features of wake and sleep states in adults?

- I always recognize these features independently without help from a supervisor
- I often recognize these features independently
- I recognize these features but need help from a supervisor
- I cannot recognize these features consistently

Q10  Are you able to recognize normal EEG features of wake and sleep states in children?

- I always recognize these features independently without help from a supervisor
- I often recognize these features independently
- I recognize these features but need help from a supervisor
- I cannot recognize these features consistently

Q11 Are you able to recognize common EEG artifacts in adults?

- I always recognize these features independently without help from a supervisor
- I often recognize these features independently
- I recognize these features but need help from a supervisor
- I cannot recognize these features consistently

Q12 Are you able to recognize common EEG artifacts in children?

- I always recognize these features independently without help from a supervisor
- I often recognize these features independently
- I recognize these features but need help from a supervisor
- I cannot recognize these features consistently

Q13 Are you able to recognize EEG patterns of status epilepticus in adults?

- I always recognize these features independently without help from a supervisor
- I often recognize these features independently
- I recognize these features but need help from a supervisor
- I cannot recognize these features consistently

Q14 Are you able to recognize EEG patterns of status epilepticus in children?

- I always recognize these features independently without help from a supervisor
- I often recognize these features independently
- I recognize these features but need help from a supervisor
- I cannot recognize these features consistently

Q15 Are you able to recognize common EEG abnormalities in adults?

- I always recognize these features independently without help from a supervisor
- I often recognize these features independently
- I recognize these features but need help from a supervisor
- I cannot recognize these features consistently

Q16 Are you able to recognize common EEG abnormalities in children?

- I always recognize these features independently without help from a supervisor
- I often recognize these features independently
- I recognize these features but need help from a supervisor
- I cannot recognize these features consistently

Q17 Are you able to recognize normal EEG variants in adults?

- I always recognize these features independently without help from a supervisor
- I often recognize these features independently
- I recognize these features but need help from a supervisor
- I cannot recognize these features consistently

Q18 Are you able to recognize normal EEG variants in children?

- I always recognize these features independently without help from a supervisor
- I often recognize these features independently
- I recognize these features but need help from a supervisor
- I cannot recognize these features consistently

Q19  Can you differentiate abnormalities in the ictal-interictal continuum from patterns that could represent status epilepticus in adults?

- I always recognize these features independently without help from a supervisor
- I often recognize these features independently
- I recognize these features but need help from a supervisor
- I cannot recognize these features consistently

Q20  Can you differentiate abnormalities in the ictal-interictal continuum from patterns that could represent status epilepticus in children?

- I always recognize these features independently without help from a supervisor
- I often recognize these features independently
- I recognize these features but need help from a supervisor
- I cannot recognize these features consistently

Q21 Can you interpret uncommon EEG abnormalities in adults?

- I always recognize these features independently without help from a supervisor
- I often recognize these features independently
- I recognize these features but need help from a supervisor
- I cannot recognize these features consistently

Q22 Can you interpret uncommon EEG abnormalities in children?

- I always recognize these features independently without help from a supervisor
- I often recognize these features independently
- I recognize these features but need help from a supervisor
- I cannot recognize these features consistently

Q23 Can you independently write an EEG report?

- I always independently generate a report without help from a supervisor
- I am often able to generate a report independently
- I need help from a supervisor to generate a report
- I cannot generate a report independently

End of Block: Default Question Block

Start of Block: Block 1

Q23 The next questions will assess your experience with reading EEGs.

Q26 How many EEGs do you estimate you have read during residency? "Read" means reviewed an EEG in its entirety, but does not need to include drafting an EEG report.

________________________________________________________________

Q27 How many EEG reports do you estimate you have written during residency?

________________________________________________________________

Q28 Since completing your pediatric epilepsy or EEG rotation, have you re-used any of the following resources?

|  | No | Yes |
| --- | --- | --- |
| Normal EEG PowerPoint |  |  |
| Abnormal EEG PowerPoint |  |  |
| Using Natus PowerPoint |  |  |
| Learningeeg.com |  |  |
| Independent EEG reading (not staffed) |  |  |
| EEG atlas |  |  |

End of Block: Block 1

Start of Block: Block 2

Q52 Thank you for completing our survey. The answer key for the objective EEG reading questions can be found in the ChildNeuroBox "EEG Curriculum" folder in the subsection "EEG Curriculum 2023." The password for the answer key document is EEGLearn2023!. You will need to download the document to enter the password.

End of Block: Block 2

**ANSWER KEY**

1. A 33-year-old woman presents to the emergency room after having a seizure. Your

workup includes a routine EEG prior to the patient leaving the emergency room. Which

of the following statements is true?

a. **The presence of focal interictal epileptiform discharges on EEG has a high positive**

**predictive value for epilepsy**

b. The absence of focal interictal epileptiform discharges on EEG will rule out epilepsy

c. If the event concerning for seizure is not captured on EEG, the test will not help predict

the patient’s risk of epilepsy

d. The frequency of focal interictal epileptiform discharges on EEG does not have

significant prognostic values

**Answer key:** The presence and frequency of interictal epileptiform abnormalities on EEG can be suggestive of an increased risk of epilepsy, even if a specific event concerning for seizure is not captured on EEG. The absence of interictal discharges does not rule out epilepsy as people with seizures may sometimes have a normal interictal EEG.

2. When is the rhythm outlined in the box typically seen on EEG?

.
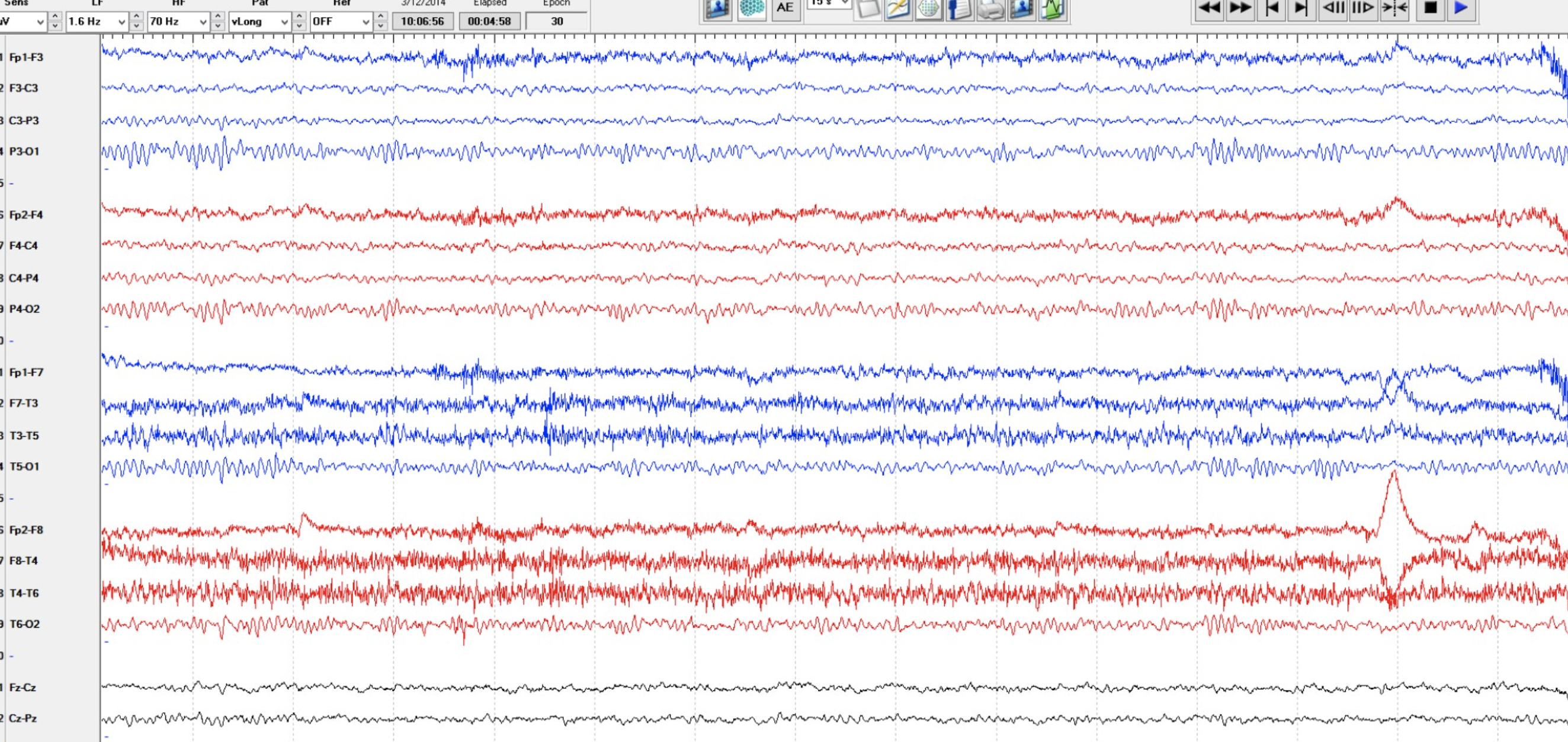


1. During drowsiness
2. With thought of moving the contralateral arm
3. **During wakefulness with eyes closed**
4. During strenuous mental tasks
5. During sleep

**Answer key:** The highlighted rhythm is the posterior dominant rhythm which is an alpha rhythm typically seen in the posterior leads with eyelid closure. It attenuates during drowsiness, sleep and strenuous mental activity so is not seen as well during these times. Mu rhythm is alpha rhythm that occurs in the central leads and attenuates with movement or thoughts of movement of the contralateral arm.

3. What is the patient most likely doing during the EEG segment below?


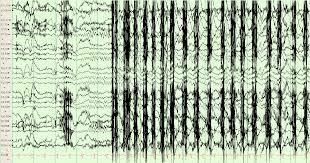


a. Having a generalized convulsion

b. Walking around the room

**c. Eating a snack**

d. Shaking their head from side to side

**Answer key:**

The EEG represents chewing artifact. It can be identified as a myogenic artifact with a predominance over the temporal leads.

4. What is the name of the rhythm seen in the box

displayed in this image?


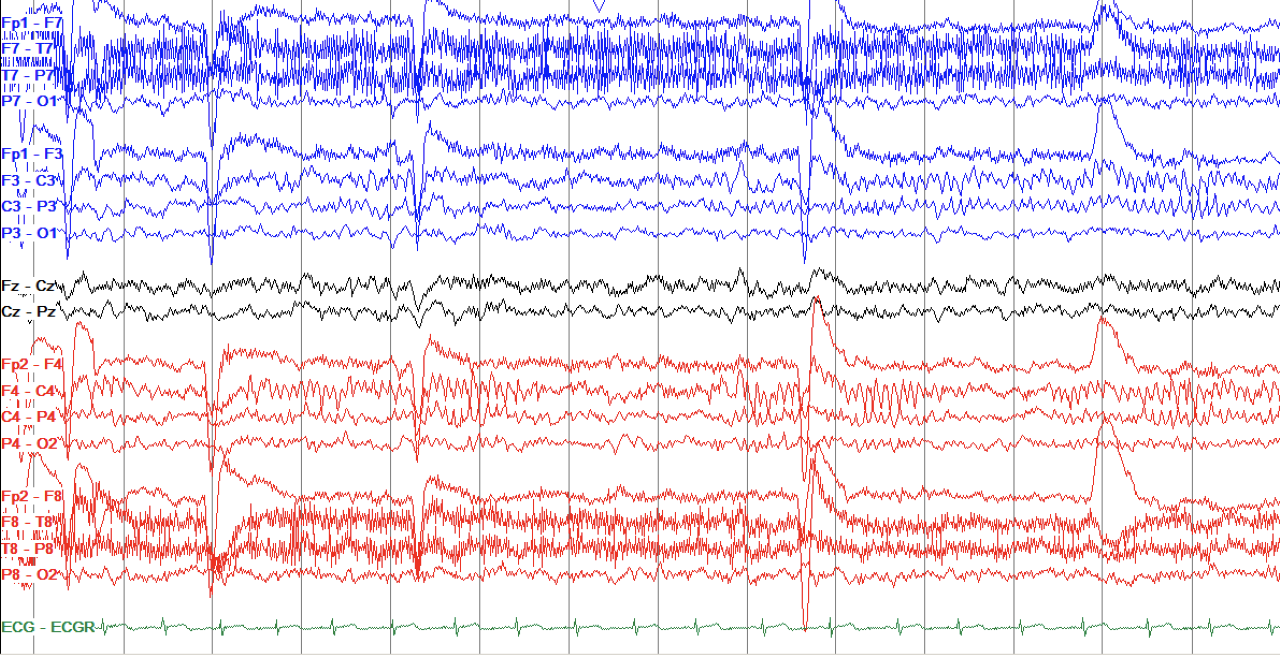


a. Alpha rhythm

b. Third rhythm

**c. Mu rhythm**

d. Posterior dominant rhythm

e. Breech rhythm

**Answer Key:** The activity seen over the right central (C4) head region here is typical Mu rhythm. The rhythm is usually in alpha range with rounded positive portion and sharpened negative portion. It represents normal sensorimotor cortex activity and can be attenuated by sensory stimulation, movement or even thought of movement of the contralateral arm.

5. In the displayed EEG, what is this box over the right temporal region highlighting?


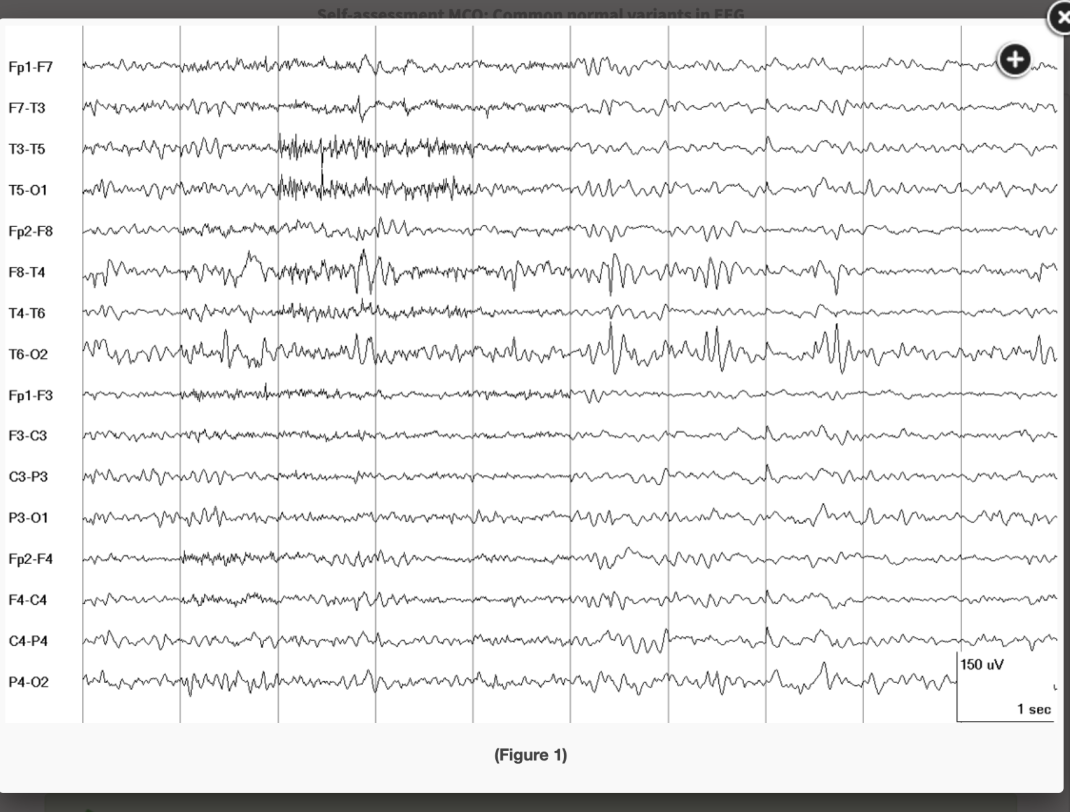


a. Mu rhythm

**b. Wicket Spike**

c. Temporal Spike

d. POSTs

**Answer key:** The sharply contoured waveforms over T4-T6 are wicket spikes. They can be distinguished from temporal spikes because they do not distort the background, and do not have an after going slow wave. They are present in drowsiness and light sleep. Mu rhythm is the idling rhythm of the sensorimotor cortex seen over central regions. POSTs are seen during drowsiness/N1 sleep and are in the occipital region.

6. How old is the patient whose normal awake EEG is pictured here?


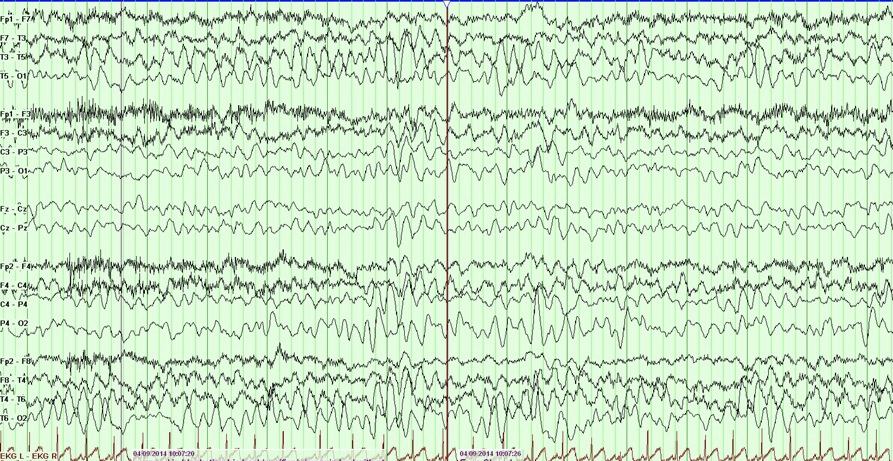


a. 2 months

**b. 10 months**

c. 5 years

d. 10 years

e. 16 years

**Answer key;** This normal EEG shows a background that is predominantly theta activity and a posterior dominant rhythm of 5 Hz. Children under age 2 months typically do not have a posterior dominant rhythm. From age 3 years and up, the posterior dominant rhythm should be 8 Hz or greater. The overall background under age 4 months is typically delta activity. The overall background from 3 years to puberty is typically alpha and theta activity, and after puberty should be alpha and beta activity. So by the features of this EEG, the child must be older than 4 months but less than 3 years - hence the correct answer of 10 months.

7. Which of the following is correct about posterior slow waves of youth?

a. They consist of beta activity within the posterior dominant rhythm

b. They are abnormal in children

c. They are mainly seen during sleep states in the occipital leads

**d. They consist of delta frequency activity within the posterior dominant rhythm**

e. They block with eye closure

**Answer key:** Posterior slow waves of youth are a normal feature of the awake EEG in children. Posterior slow waves of youth consist of delta activity within the posterior dominant rhythm and therefore become attenuated with eye opening and are augmented with eye closure.

8. Which of the following is a component of N1 sleep?

**a. POSTs**

b. Sleep spindles.

c. K-complexes.

d. High-voltage polymorphic delta.

**Answer key:** POSTs are seen during N1 sleep and are defined as positive deflections in the posterior leads. Sleep spindles and K complexes do not appear until N2 sleep and high-voltage polymorphic delta is seen in slow wave sleep.

9. The finding below, appears first in which stage of sleep?


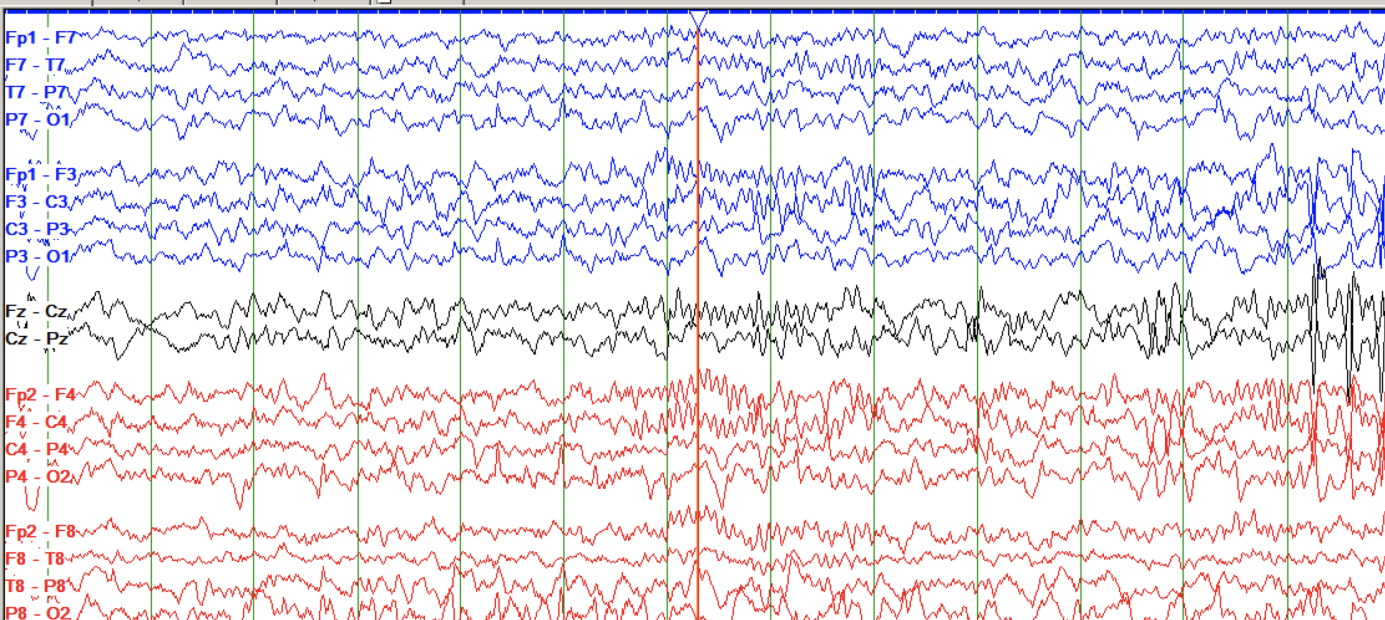


a. Drowsiness

b. N1 Sleep

**c. N2 Sleep**

d. N3 Sleep

e. REM Sleep

**Answer key:** Sleep spindles are highlighted in the box above and are defining features of N2 sleep. They are identified as 12-16hz activity in frontocentral regions. Sleep spindles are likely generated by synchronous thalamo-cortical oscillations during sleep. POSTS and vertex waves can be seen in N1 sleep, and all of these features can be seen in slow wave N3 sleep. REM sleep does not usually contain sleep transients.

10. At what age do sleep spindles first appear?

a. Birth

**b. 2 months of age**

c. 2 years of age

d. 4 years of age

e. 10 years of age

**Answer key:** Sleep spindles typically emerge at age 2-3 months and are often asynchronous with a long duration (up to 10-15 seconds) in the first year of life. By age 18 months, sleep spindles are typically shorter duration (1-5 seconds) and more synchronous.

11. What direction is the patient looking within the area highlighted by the box?


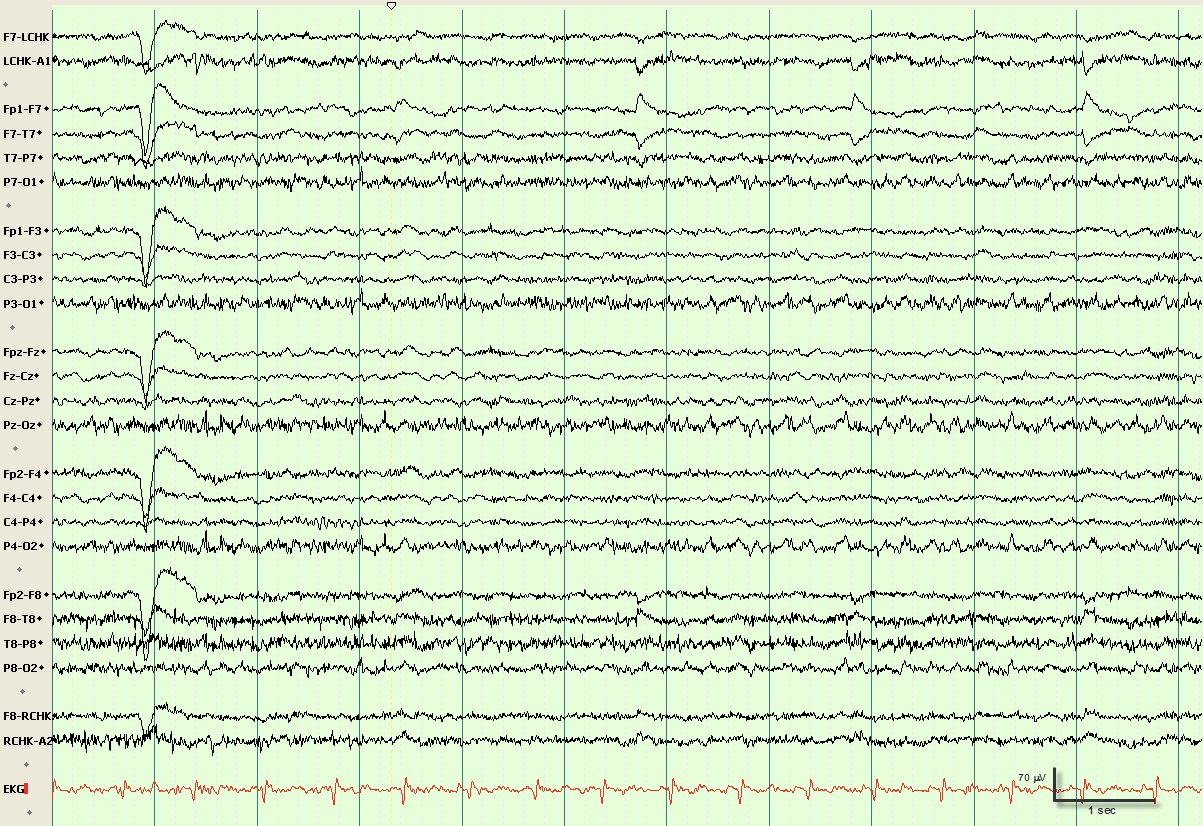


a. Right

b. Up

**c. Left**

d. Down

Answer key: The patient is looking left in the highlighted box. The best leads for lateral eye movement are F7 (left) and F8 (right). The cornea is more positive than the retina. When looking left, F7 sees more positivity from the cornea which gives a positive phase reversal on a bipolar montage. F8 sees more negativity from the retina giving a negative phase reversal on a bipolar montage.

12 What is the likelihood that this EEG pattern is consistent with non-convulsive status

epilepticus (NCSE) in a patient with no clinical correlate to this pattern?


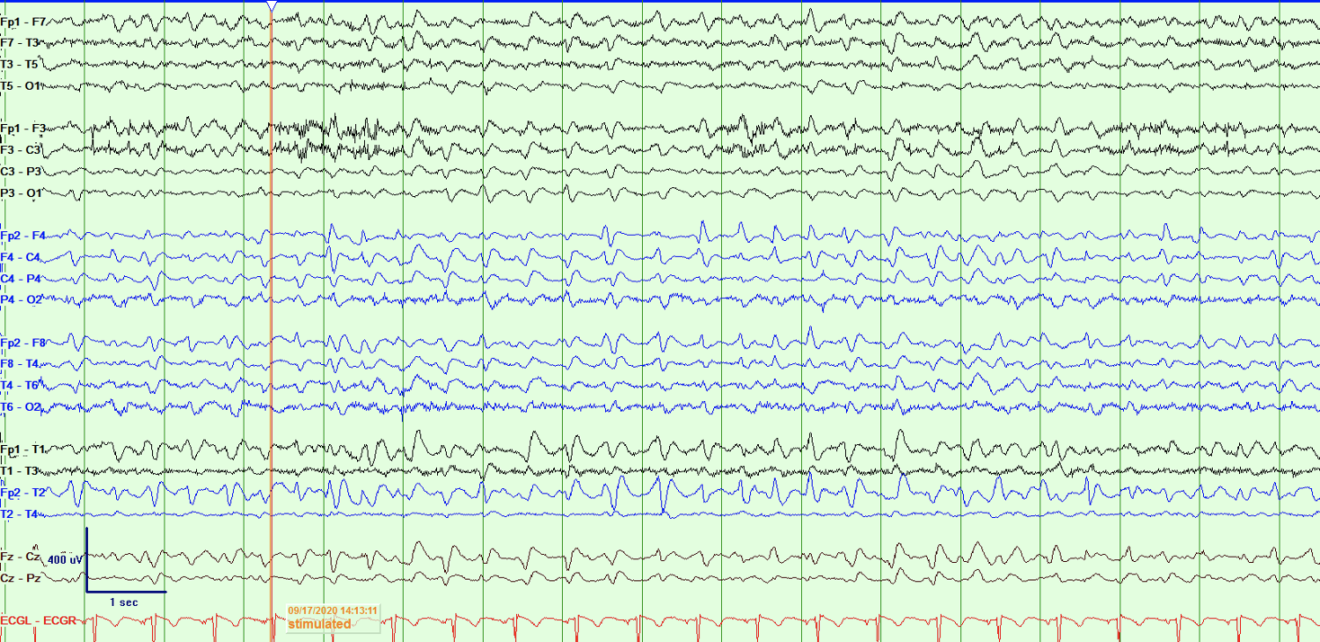


a. **The EEG is probably not consistent with NCSE because the rhythmic**

**discharges are slower than 2.5hz and do not evolve**

b. This EEG is probably not consistent with NCSE because the rhythmic discharges are

triphasic appearing

c. This EEG is probably NCSE because there are rhythmic discharges greater than 2.5hz

d. This EEG is probably NCSE because this pattern emerged after stimulation of the

patient

**Answer key:** Answer A is correct because typically discharges should reach at least 2.5hz to be concerning for NCSE. Answer B is incorrect because some seizures can have discharges of triphasic morphology. Answer C is incorrect because rhythmic discharges <2.5 Hz as seen here are not necessarily ictal. Answer D is incorrect because stimulation-induced rhythmic discharges are not necessarily ictal.

13. A 30-year-old man with a known focal cortical dysplasia (FCD) is admitted to the Neuro-

ICU confused, with very frequent clusters of rhythmic jerking of his left face and arm. He is unable to follow commands. See EEG image attached. How would you best classify this

status presentation according to the ILAE Classification System for Status Epilepticus?


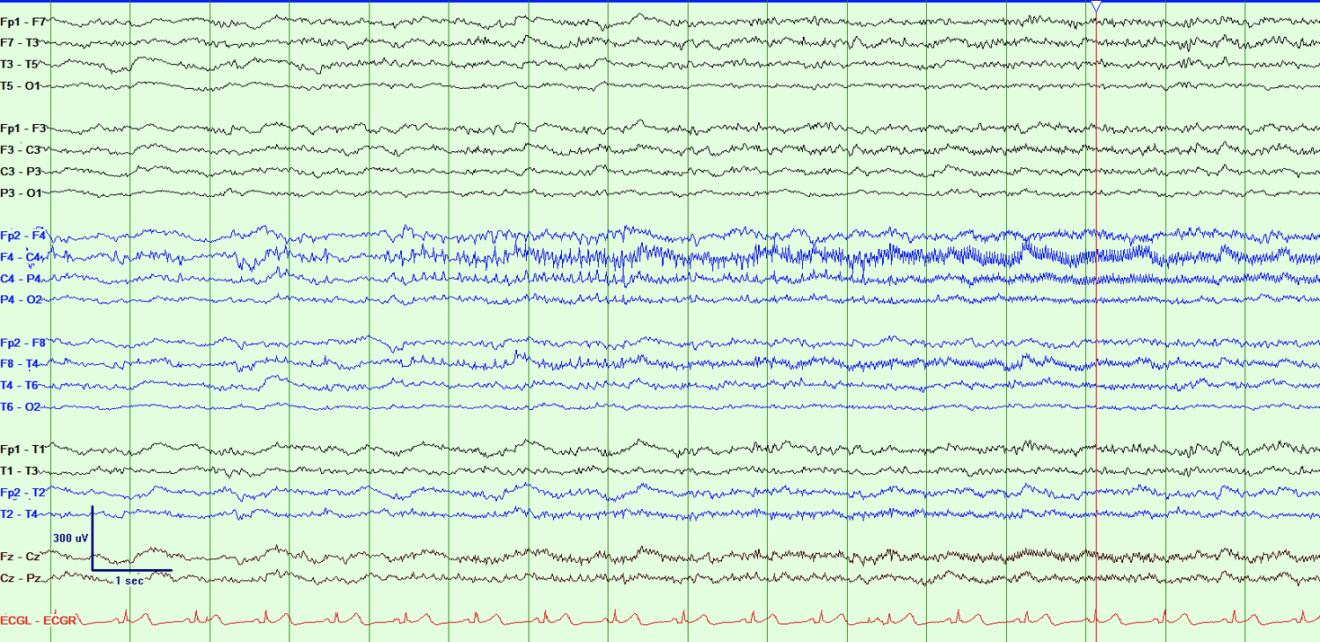


a. Convulsive status epilepticus

b. Non-convulsive status epilepticus

c. Focal non-motor status epilepticus with impaired consciousness

**c. Focal motor status epilepticus with impaired consciousness**

d. Focal motor status epilepticus with secondary generalized convulsive status epilepticus

**Answer key:**

ILAE Classification System of SE provides a framework for clinical diagnosis, investigation, and therapeutic approaches for each patient. One way it classifies is by semiology which can be divided into motor and nonmotor status. Motor status includes convulsive or tonic-clonic status which does not sound consistent with the above case given the semiology of the seizures is focal twitching of the left face and arm. He has motor symptoms so this would not be considered focal non-motor status and he has clear clinical motor seizures which is less consistent with non-convulsive status epilepticus. His EEG primarily shows evolving focal seizure over the right fronto-central region (more prominent over the anterior quadrant of the EEG) without generalization to other leads suggestive of secondary generalized convulsive status epilepticus.

14 What is a “breach rhythm”?

a. A focal increase in the amplitude of low-frequency activity.

b. A focal decrease in the amplitude of low-frequency activity.

**c. A focal increase in the amplitude of high-frequency activity.**

d. A focal decrease in the amplitude of high-frequency activity.

**Answer key: Breach** rhythm is high amplitude, high-frequency, often spiky and irregular appearing activity seen in a region of skull opened due to brain surgery.

15. There is a 45-year-old woman who has the EEG noted below. What abnormality do you

see in this EEG?


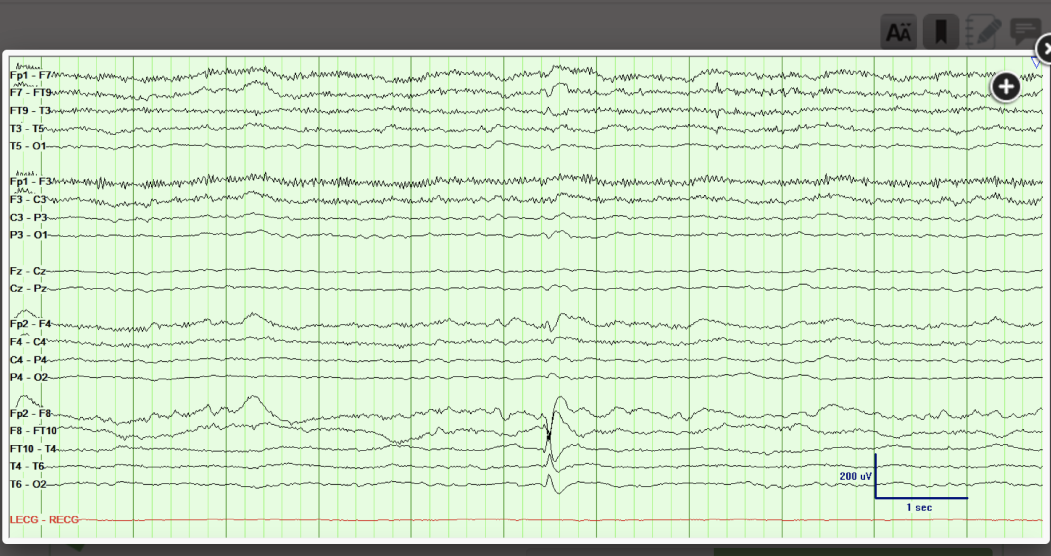


**a. Temporal epileptiform discharge**

b. Occipital epileptiform discharge

c. Frontal epileptiform discharge

d. Central epileptiform discharge

e. Rolandic epileptiform discharge

**Answer key;** Epileptiform discharges are classified by their location. This is a bipolar montage that shows a phase reversal at FT10, which registers epileptiform discharges in the anterior temporal region. There is no phase reversal in any of the other locations listed in the other choices.

16. What does the presence of the following pattern below suggest?


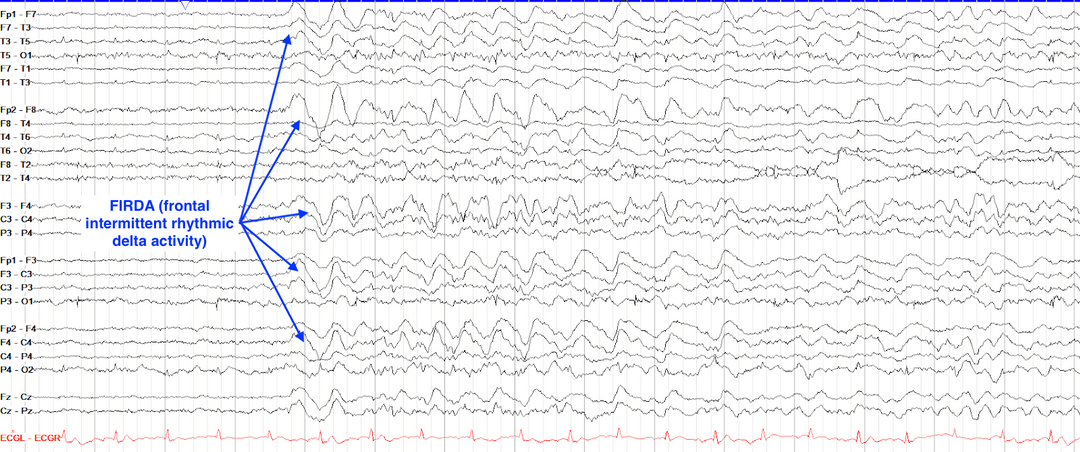


1. Increased risk for seizures
2. **Nonspecific cerebral dysfunction**
3. Nothing: this is artifact from frequent eye blinks
4. This pattern is concerning for status epilepticus

**Answer key:** This EEG is showing generalized rhythmic delta activity (GRDA) in a pattern previously called FIRDA (frontal rhythmic delta activity). This finding is primarily associated with cerebral dysfunction. Temporal rhythmic delta activity (TRDA) is more associated with increased risk of seizure. Eye blinks would have a positive, not negative phase reversal in the frontal leads.

17. You are treating a 20-year-old woman whose EEG shows the findings below. What is her

most likely diagnosis?


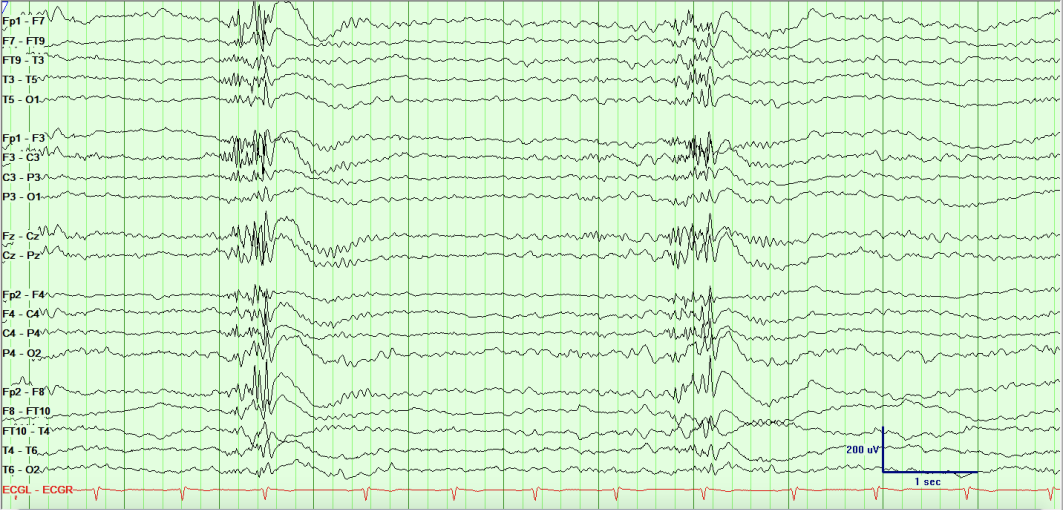


**a. Juvenile myoclonic epilepsy**

b. Childhood absence epilepsy

c. Lennox-gastaut syndrome

d. West syndrome

e. Tonic epilepsy

**Answer key:** The EEG in patients with Juvenile Myoclonic Epilepsy often shows generalized polyspike and wave discharges, which are demonstrated in this EEG. In Childhood Absence Epilepsy, the EEG will typically show bursts of 3Hz generalized spike and wave discharges. In Lennox-Gastaut Syndrome, the EEG will show slow spike and wave discharges, typically in bursts of 1.5-2.5Hz. The EEG in West Syndrome often shows hypsarrhythmia and electrodecrement. The EEG in Tonic Epilepsy can show periods of generalized paroxysmal fast activity

18. Which of the following lists the typical settings in Natus for low frequency filter, high

frequency filter, notch and sensitivity?

a. 0.1, 80, 60, 7

**b. 1, 70, 60, 7**

c. 0.5, 80, 70, 10

d. 2, 70, 60, 5

**Answer key:** As above, the typical low frequency setting is 1, high frequency is 70, notch is 60 and the sensitivity starting point is 7.
